# Using optical skyrmions to assess vectorial adaptive optics capabilities in the presence of complex aberrations

**DOI:** 10.1126/sciadv.adv7904

**Published:** 2025-10-03

**Authors:** Yifei Ma, Zimo Zhao, Yuanxing Shen, Binguo Chen, An Aloysius Wang, Yuxi Cai, Ji Qin, Runchen Zhang, Yunqi Zhang, Jiahe Cui, Bangshan Sun, Jiawen Li, Yuzhu Shi, Liangyu Deng, Honghui He, Lin Luo, Yonghong He, Yun Zhang, Ben Dai, Daniel J. Royston, Steve J. Elston, Stephen M. Morris, Martin J. Booth, Chao He

**Affiliations:** ^1^ Department of Engineering Science, University of Oxford, Parks Road, Oxford OX1 3PJ, UK.; ^2^ Guangdong Research Center of Polarization Imaging and Measurement Engineering Technology, Institute of Biopharmaceutical and Health Engineering, Tsinghua Shenzhen International Graduate School, Tsinghua University, Shenzhen 518055, China.; ^3^ College of Engineering, Peking University, Beijing 100871, China.; ^4^ Key Laboratory of Archaeological Sciences and Cultural Heritage, Chinese Academy of Social Sciences, Beijing 102488, China.; ^5^ Department of Statistics, The Chinese University of Hong Kong, Shatin, HK SAR, China.; ^6^ Nuffield Division of Clinical Laboratory Sciences, Radcliffe Department of Medicine, University of Oxford, Oxford, UK.; ^7^ Department of Pathology, Oxford University Hospitals NHS Foundation Trust, Oxford, UK.

## Abstract

With the growing use of optical polarization in applications ranging from communications to medical diagnoses, adaptive correction of complex vectorial aberrations in optical systems has become an increasingly important area of research. However, research to date has focused primarily on phase and retardance aberrations, whereas another major source of aberration—diattenuation—remains largely unexplored. Unlike the others, diattenuation affects intensity in addition to phase and polarization, limiting the intrinsic correction capability of adaptive systems. In this work, we propose the use of optical skyrmions to probe diattenuation-aberrated systems and provide metrics that characterize the performance of vectorial adaptive optics (V-AO), with theoretical and experimental validations. Based on the probed results, we demonstrate V-AO correction under real-world aberrations for complex media imaging and analyze correction strategies to optimize measurements in aberrated polarimetric systems. This work paves the way for high-dimensional aberration correction, introduces a previously unidentified use of optical skyrmions, and provides insights that will aid the development of vectorial measurement systems.

## Teaser

Optical skyrmions are used as probes to access vectorial adaptive optics performance with complex aberrations.

## INTRODUCTION

Adaptive optics (AO) is a widely used and powerful tool that has been adopted in numerous optical systems ranging from applications in astronomy (*1*) to microscopy (*2*, *3*), aerospace engineering (*4*), and communications (*5*). The technique is usually thought of as the feedback correction of phase aberrations (*6*), but the underlying concept can be extended to correct for vectorial counterpart, which contain both phase and polarization distortions. This is notable as there are various optical systems where the existence of vectorial aberrations such as retardance and diattenuation also has detrimental effects (*7*–*10*). Such aberrations induce errors in the state of polarization (SoP) and introduce extra (dynamic and geometric) phase distortions, leading to imperfect interference at the focus and affecting the resolution of the system (*9*). The correctness of vectorial information is also of huge importance in many applications such as label-free cancer boundary detection through polarimetric measurements (*10*–*13*) and the noninvasive analysis of material samples (*14*). Without vectorial aberration correction, it is challenging to achieve the necessary information correctness in aberrated Stokes vector and Mueller matrix microscopy needed for a wide range of fields.

**Fig. 1.**
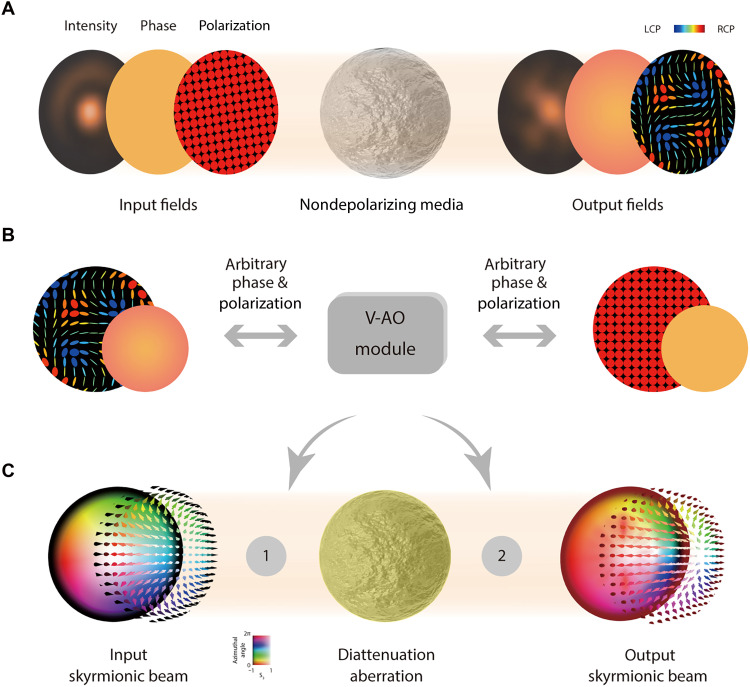
Nondepolarizing media, V-AO correction module, and schematic of the V-AO correction strategy for a diattenuation aberration. (**A**) Illustrations of how nondepolarizing media affect the spatial distributions of intensity, phase, and polarization fields. Retardance in nondepolarizing media affects phase and polarization, whereas diattenuation affects phase, polarization, and intensity (*9*, *15*, *61*, *62*). The spatially varying distributions of intensity, phase, and polarization fields before and after passing through the nondepolarizing media are shown. Circular planes represent the beam’s spatial profiles in each of the three domains. LCP, left-handed circular polarization; RCP, right-handed circular polarization. (**B**) The V-AO module enables arbitrary phase and SoP conversion. This capability is essential for correcting spatially varying vectorial aberrations, such as phase and retardance aberrations, that arise in nondepolarizing media. (**C**) The V-AO correction strategy toward diattenuation aberration is illustrated, with the V-AO module positioned before (location 1) or after (location 2) the aberration. It also illustrates the Stokes fields of a typical Néel-type skyrmion as it passes through this diattenuation aberration. Color is used to represent the azimuthal angle on the PS, and saturation is used to represent height through the paper [similar to ref. (*34*)].

Vectorial adaptive optics (V-AO) involves the use of suitable corrections to negate the effects of vectorial aberrations in nondepolarizing media, where phase, retardance, and diattenuation aberration may exist (see Fig. 1A) (*15*, *16*). It uses polarization-sensitive AO devices such as spatial light modulators (SLMs) to manipulate the SoP and phase in a spatially varying manner (*16*–*22*). Such devices with a large dynamic range have been shown to effectively compensate for aberrated phase and SoPs through precorrection methods under certain conditions (*9*). For phase aberration, AO devices can always work either in pre- or postcorrection within their dynamic range (*2*); the same can be said for retardance aberrations, provided that there are sufficient correction degrees of freedom so that SoP field conversion can be attained (*16*–*22*). Because phase and retardance aberrations have been readily compensated by existing strategies across various scenarios in prior works, we focus here solely on diattenuation in the analysis of nondepolarizing media.

When it comes to diattenuation aberration, the decrease in intensity as light passes through the system intrinsically limits its amenability to correction. The errors introduced by a perfect polarizer, to take a trivial example, would be impossible to correct. This highlights the need to devise metrics that characterize diattenuating systems on the basis of their potential correctability. Doing so enables a better understanding of the degree of correction that can be achieved, considering given important physical parameters of the setup such as camera sensitivity. One way such a metric can be formulated is to consider the mapping that the system induces on the Poincaré sphere (PS). This motivates us to introduce nontrivial structured light, in the form of an optical skyrmion, which simultaneously has every possible SoP as a means to probe and characterize a system.

The skyrmion (*23*) is a unified model to describe fundamental particles. Since its conception, the topological nature of the magnetic skyrmion has driven investigations into its use as potential information carriers, and such structures have been touted for their potential to revolutionize ultradense data storage (*24*–*28*). Recently, optical skyrmions have been observed in evanescent waves, where their topological properties are carried through a spatially varying polarization field (*29*–*31*). Since then, the generation of optical skyrmions has been achieved in paraxial vector beams through a variety of different techniques (*31*–*37*). The topological properties of the skyrmion along with associated applications still offer large scopes to be explored.

Skyrmions are characterized by their topological number (*38*, *39*) [skyrmion number (SN)] defined as

$$\text{SN}=\frac{1}{4\pi }\iint_{\sigma }n\cdot \left(\frac{\partial n}{\partial x}\times \frac{\partial n}{\partial y}\right)\text{dxdy}$$ (1)

where n(x,y) represents the vector field that describes the two-dimensional skyrmion, and σ represents the confined region.

In this work, we harness the SN as a global, quantitative metric, and a Néel-type skyrmionic beam (*34*) as a probe for characterizing nondepolarizing optical systems and understanding the correctability of the V-AO system. On the basis of probing results, we performed V-AO correction experiments on complex biomedical and archaeological samples to assess correction performance under different aberrations. We furthermore elaborate on a V-AO correction methodology for optimizing measurements in aberrated polarimetric microscopes. This study explores a quantitative analysis of the V-AO correction strategy for general nondepolarizing aberrations. Our work opens avenues to the advanced use of the optical skyrmion in the context of V-AO, with broad implications for the future of sensing and imaging technologies.

## RESULTS

### V-AO for diattenuating systems

A diattenuator has two different absorption ratios for two eigen polarization states (*40*); it, in effect, attenuates the intensity of one eigenstate more than the other. Consequently, not only will there be a power reduction when light passes through a diattenuator but also a change of SoP. Unlike phase or retardance aberrations, which can be fully compensated given a sufficient correction dynamic range or degrees of freedom (as they did not change polarization entropy and therefore theoretically did not affect the correctability of AO devices), diattenuation can reduce portions of the beam’s intensity below the camera’s detection threshold. This intensity limitation fundamentally restricts correctability and does not occur when only phase or retardance is involved. Diattenuation can be induced from various common optical elements including through Fresnel’s effects due to reflection and refraction and the intrinsic polarization properties of biomedical tissues or materials samples (*8*–*13*). Note that an arbitrary diattenuator is characterized by three parameters: its extinction ratio ( E ), transmissive axis shape ( S ), and axis orientation ( θ ), with comprehensive mathematical details provided in note S1.

In a previous work, V-AO correction module was effectively realized by a cascade of two SLMs and a deformable mirror (DM) (*9*). To achieve arbitrary-to-arbitrary phase and arbitrary SoP to arbitrary SoP conversion (ATA), a V-AO module comprising four SLMs (or three SLMs and a DM) is also proposed (*14*, *17*–*19*) (see Fig. 1B). Note that because phase, as a scalar quantity, can be arbitrarily modulated using a single device, thus our experiments in this work mainly adopt the three-SLM configurations to specifically target polarization control. The versatility of ATA devices enables their broad applications across many different scenarios, including the illumination and detection arms of modern Stokes/Mueller microscopes (*10*) that require specific uniform SoPs for effective sample illumination and analysis (*41*, *42*).

In V-AO, the matrix reciprocity issue is crucial, particularly the placement of AO devices relative to aberrations influences system performance. With ATA ability, assuming no intensity loss, an AO module placed after the aberration (see Fig. 1C, location 2) can always correct for the system, barring complete intensity attenuation. However, positioning the AO device before the aberration (see Fig. 1C, location 1), which is required by the precorrection systems and associated applications, will introduce complexity. This is because a diattenuator may attenuate certain SoPs in the output field, which cannot be recovered via precorrection, thus limiting the aberration correction ability of V-AO even with perfect ATA. For instance, one can easily show that if the degree of polarization of the incident light is smaller than the diattenuation of the system, then the transformation of the PS induced by the diattenuating element is not surjective (*37*), and therefore there exist SoPs that can never be present in the output.

### Characterizing the correctability of diattenuating systems using optical skyrmions

By harnessing the unique characteristics of optical skyrmionic beams, whose SoP distribution spans the entire PS, as well as the SN, we can use spatially uniform samples to define the correction boundary of the V-AO system through a single-shot spatial measurement rather than sequential temporal measurements (see note S2). By generating such beams before an aberrating element and subsequently measuring the beam profile, we can effectively determine how the element alters SoPs, thus evaluating the retained V-AO correction ability. It is worth noting that, in most practical vectorial measurement scenarios, the analysis mainly hinges on intensity measurements, with scientific cameras commonly serving as intensity detectors. Throughout this work, we take the camera’s sensitivity into account to eliminate regions with lower intensity levels that fall below the detection threshold. This step is crucial for determining the SoPs that can be reliably characterized.

Diattenuators can attenuate the intensity of polarized light to such an extent that their signals fall below the detector’s noise floor, resulting in measurement errors or undetectable data. To quantify this, a specific intensity value *F*, calibrated in accordance with the detectors’ signal-to-noise ratio, is applied to filter out the output field, with any signals beneath this threshold deemed undetectable. For a unit Néel-type skyrmionic incident beam, this exclusion corresponds to a spherical cap on the PS surface, with its radius determined solely by the given intensity criterion *F* and extinction ratio *E* of the diattenuator, as elaborated in note S2.

It is intuitive that, after modulation by a diattenuator, if the beam still serves as a single unit skyrmionic beam (with SN = 1), it implies the preservation of all SoPs in the output. Hence, an AO device can do precorrection to correct the errors introduced by any such aberration. Conversely, a deviation from SN = 1 signifies that the output beam no longer contains all the SoPs, suggesting that there exist certain SoPs that cannot be precorrected by V-AO. Therefore, the SoP diversity in the output field effectively reflects the V-AO system’s capability to enable effective aberration compensation. In particular, the change in SN serves as a quantitative measure for the loss of SoPs through the diattenuator. Beyond the correctability of V-AO, such an evaluation is important for many applications, such as advanced dipole orientation–based fluorescence super-resolution microscopes (*3*) or Stokes vector/Mueller matrix microscopes (*10*), where the ability to generate arbitrary SoPs is essential for effective illumination and analysis. We note that this approach does not conflict with the topological protection properties of optical skyrmions under diattenuation as defining the integration region through intensity filtering already breaks the field continuity, and thus the SN is expected to change (*37*); Therefore, we define the new integration region by excluding the portion of the beam that falls below the intensity threshold, in accordance with the criterion described above.

**Fig. 2.**
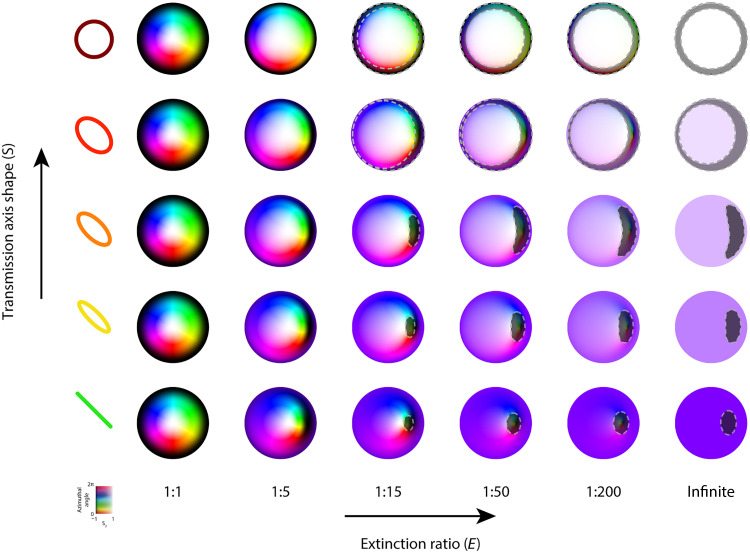
Output skyrmionic fields after being modulated by different diattenuators. Illustration of how different extinction ratios (represented on the *x* axis) and the transition of the transmissive axis shape from linear to right-hand circular (depicted on the *y* axis) influence the Néel-type skyrmionic beams. The orientation of transmissive axis is consistently maintained at 45° throughout the work. The highlighted shaded area (dark gray area) indicates regions excluded by applying an intensity filter threshold of F=0.1 , illustrating the impact of diattenuation on the detectability of certain field regions.

To investigate the correction ability of V-AO for compensation of arbitrary diattenuation, we used such a Néel-type optical skyrmionic beam as input field and conducted simulations to evaluate the change in SN after passing through a diattenuator. We keep the orientation θ of the transmissive axis fixed at 45° throughout this work as it does not affect the loss of SoPs. Figure 2 illustrates the output fields obtained from the input beam interacting with different diattenuators. The *x* axis of Fig. 2 represents the different extinction ratios ( E ) of the diattenuators, whereas the *y* axis represents different axis shape ( S ). See details in note S1. The observed trend shows an increasing homogeneity in the output fields with rising E values, reaching completely homogeneous when E approaches infinity. A similar effect can be observed along the *y* axis with the increasing ellipticity of S.

**Fig. 3.**
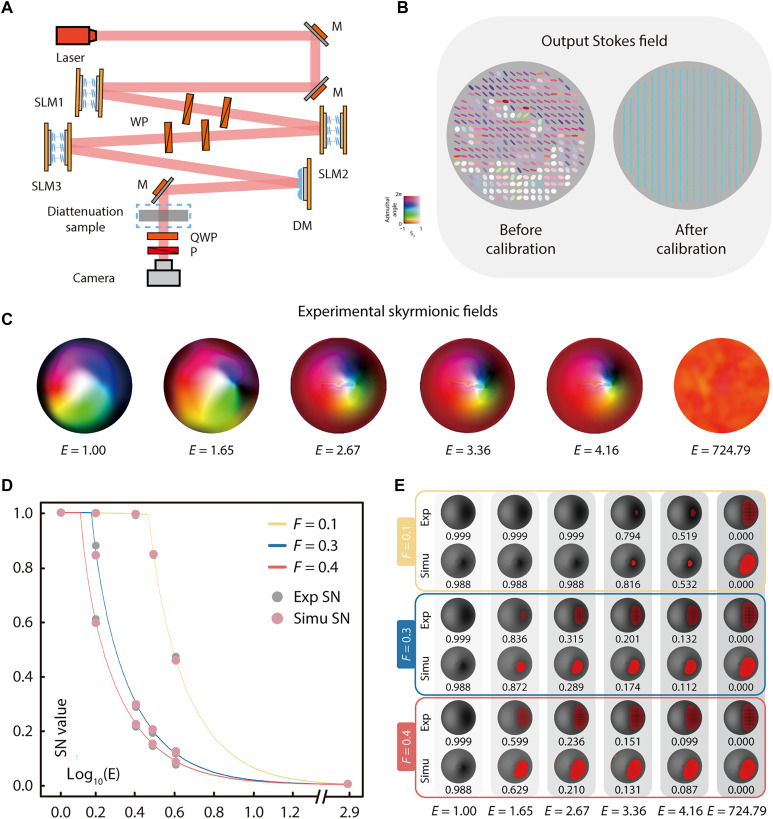
Characterization of output skyrmionic fields and SN values under different diattenuation aberration configurations. (**A**) Experimental setup comprising a skyrmionic beam generator and a Stokes polarimeter. A He-Ne laser (Melles Griot, 05-LHP171, 632.8 nm) serves as the light source. The beam is modulated by a cascade of three SLMs (Hamamatsu, X10468-01) to generate the desired SoP field, with a DM (Boston Micromachines Corporation, Multi-3.5) used to compensate for initial phase aberrations in the system. A waveplate assembly (WP; Thorlabs, WPH10M-633) sets the axes of two SLMs at a 45° angle. The modulated beam then passes through the diattenuation sample and is analyzed by a Stokes polarimeter comprising a QWP (Thorlabs, WPQ10M-633), a polarizer (P; Thorlabs, GL10-A), and a camera (Thorlabs, DCC3240N). (**B**) Output Stokes fields before and after SLM calibration. Different ellipses and colors represent different Stokes vectors, as illustrated in Fig. 1C. (**C**) Experimental skyrmionic fields after propagation through diattenuations with extinction ratios E=1,1.65,2.67,3.36,4.16, and 724.79 . Notably, the first five configurations were achieved using single or cascaded weak diattenuators (i.e., beam splitters), whereas the final configuration used commercial polarizers to simulate strong diattenuators (theoretically approaching infinite E values). (**D**) Simulated relationship between SN and E , evaluated under three intensity thresholds: F=0.1,0.3 , and 0.4 . SN is calculated over regions with intensity greater than F . A logarithmic scale (base 10) is used to encompass a broad range of diattenuation values. (**E**) Simulated and experimental output intensity images for each threshold F . The top and bottom rows show simulated and experimental results, respectively. Red areas indicate regions excluded by the threshold F . Corresponding SN values are shown below each image.

We then further conducted a proof-of-concept experiment to validate that the SN can be used to evaluate the V-AO correction ability for diattenuation aberrations. An incident unit skyrmionic beam was generated via a cascade of SLMs as shown in Fig. 3A [see ref. (*14*) and Methods for a detailed description of optical skyrmion generation]. The calibration result of SLMs is shown in Fig. 3B (see note S3 for details), demonstrating the precise pixelated control of SLMs.

The experiment proceeded as follows. First, after initial SLM calibration, the correct phase patterns were applied to each SLM to generate the target skyrmionic beam. Second, the diattenuation configuration (see note S4 for polarimetric characterization and additional comparative results) was placed after the DM. Third, a Stokes polarimeter was used to measure the output SoP field after the beam passed through the diattenuation. This process was repeated for different diattenuation configurations. We then calculated the output Stokes/skyrmionic fields as well as their SN values, and the results are given in Fig. 3C (see note S4 for simulation and experimental results comparison, together with analysis of the residual errors). Figure 3D presents three simulated curves on the basis of different intensity thresholds, illustrating the changes in SN as E increases. The experimental and simulated SN values are also illustrated. This demonstrated that the change of SN can quantitatively indicate the diminishing correction ability of the V-AO system within our framework.

Note that the sudden drop in the SN observed in Fig. 3D is primarily attributed to the implementation of the intensity filter threshold. As the diattenuation value increases, the attenuation of light intensity becomes more pronounced, resulting in certain SoPs in the output field falling below this threshold, which are marked red in Fig. 3E. These SoPs are consequently excluded from the analysis, leading to a sharp decline in SN.

As the homogeneity of the Stokes vector field increases, the correction ability of V-AO diminishes due to an augmented loss of SoPs. This shows that, within any V-AO framework using precorrection strategies for existing diattenuation, there always exist SoPs that remain inherently uncorrectable via current V-AO techniques. Consequently, the correction ability toward diverse diattenuations can be, in effect, quantified via the SN, establishing a critical connection between V-AO correction ability and given diattenuator through a single metric. Such a relationship and quantitative descriptions are important as they potentially provide a lookup table, or a reference framework, to enhance the practical application of the V-AO system. For instance, the performance of advanced polarization sensing systems used in biomedical research is susceptible to diattenuation aberration due to light propagation through the sample (*43*). The correction ability can be derived by analyzing the SN value, and the optical system can be corrected accordingly (apply V-AO directly or first use passive compensation to precondition the system for possible V-AO operation), enhancing the efficiency.

### V-AO–assisted polarimetric measurement

Stokes vector and Mueller matrix microscopy are pivotal techniques for acquiring vectorial information and are valuable for material characterization and pathological diagnosis (*10*–*12*, *44*–*55*). The accuracy of vectorial information is one of the core aspects of Stokes-Mueller polarimetry but can be significantly affected by vectorial aberrations, including phase, retardance, and diattenuation. Phase and retardance can be effectively corrected by using an existing AO toolbox because they do not intrinsically limit the correction ability of the AO system within its dynamic range. However, diattenuation introduces unique challenges due to its impact on intensity in addition to phase and SoP. Despite diattenuation being common in optical components, such as beam splitters, conventional calibration methods have not addressed this problem.

In this section, we first demonstrate the effectiveness of V-AO, as probed by the optical skyrmionic field, in correcting diattenuation aberrations. We selected two spatially varying sample of high practical significance—specifically, biomedical and archaeological samples. The biomedical sample is a bone marrow trephine from a myelofibrosis case, where vectorial information captures fibrotic structural changes that are critical for pathological analysis, clinical staging, and precise treatment planning (*14*). The archaeological sample is red pigment extracted from cinnabar used in China during the Qin and Han dynasties, the vectorial analysis of the cinnabar is essential for tracing the mineral’s provenance (*14*).

**Fig. 4.**
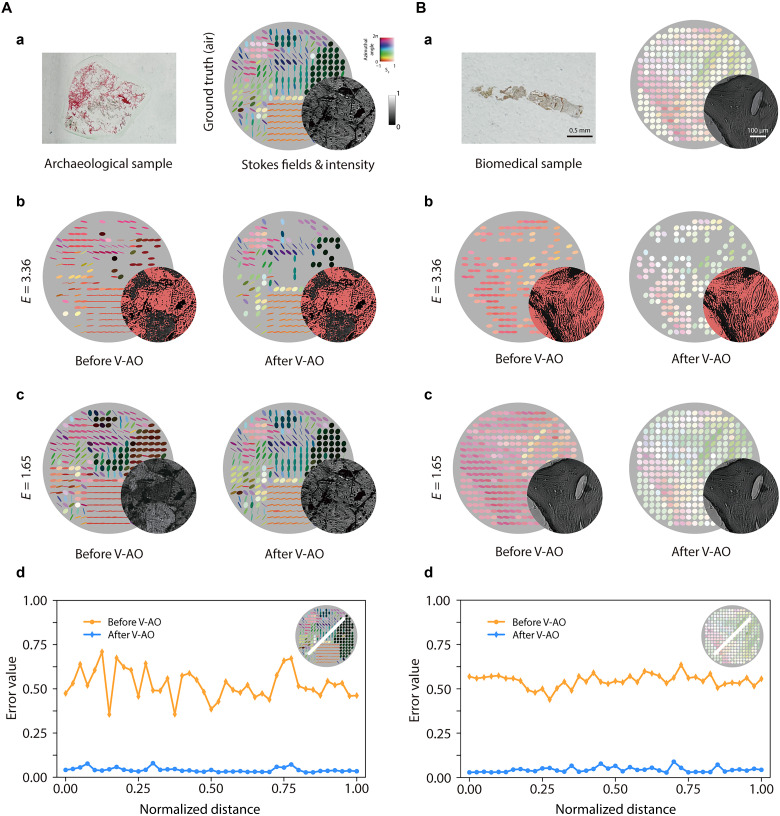
Diattenuation correction in complex media via V-AO. Stokes vector fields, intensity images, and corresponding quantitative analysis of (**A**) a biomedical and (**B**) an archaeological sample. For each sample, (a) presents the real-world picture and ground truth reference field; (b) shows results under a severe diattenuation aberration ( E=3.36 ) before and after V-AO correction; (c) illustrates the corresponding results for a moderate diattenuation aberration ( E=1.65 ); and (d) quantifies the V-AO correction performance by plotting the matching error between the corrected SoPs and the ground truth (which is evaluated by their Euclidean distance) along a selected cross section of the field with a moderate diattenuation. Regions where SoPs fall below the detection threshold are indicated by red-marked areas in the intensity image.

The procedure proceeded as follows: First, to obtain the ground truth of the samples’ vectorial information, we used a circular SoP illumination and record the polarimetric images of the samples without any induced diattenuation (*41*), with the results shown in Fig. 4 [A(a) and B(a)]; second, to validate the performance of the V-AO system under nonrecoverable aberration, a diattenuation with E=3.36 is introduced. The output Stokes fields before and after V-AO correction are shown in Fig. 4 [A(b) and B(b)]. Under this severe diattenuation, intensities of certain SoPs (masked in red) were attenuated below the detection threshold, resulting in unrecoverable SoPs, thus revealing the intrinsic correction limit of the V-AO system; third, the aberrated and corrected output Stokes field after passing through a diattenuation with E=1.65 (within the correction boundary) is recorded. As shown in Fig. 4 [A(c) and B(c)], V-AO effectively restored the Stokes fields; last, to further quantify the correction performance, we evaluated the matching error between corrected output and the ground truth SoPs by calculating the Euclidean distance between them (*14*, *16*, *56*), with results plotted in Fig. 4 [A(d) and B(d)]. See note S5 for additional quantitative analysis.

These results illustrate that, once the correction boundary of V-AO is understood, our pixelated V-AO architecture can effectively achieve aberration correction, demonstrating potential to real-world imaging scenarios where SoP fidelity is required—such as in complex or spatially varying samples affected by diattenuation.

As an extension of V-AO beyond traditional correction, we then briefly explore how the SN can also serve as an indicator of the performance of polarimetric imaging under diattenuation, particularly by guiding the selection of optimal measurement configurations, with V-AO implemented in the analyzing arm to control the detection states. For Stokes vector polarimetry, the configuration typically involves a polarization state analyzer (PSA), whereas Mueller matrix polarimetry setups incorporate a polarization state generator (PSG) and a PSA. A minimum of N ( N=4 for Stokes vector and 16 for Mueller matrix) independent SoPs is required to reconstruct the polarization information. The optimization approaches for both PSG and PSA in measurement process are analogous (*41*). We first concentrate on the analysis of the PSA as a case study.

To evaluate the performance of the PSA module, various metrics have been introduced, including condition number (CN) (*57*), equally weighted variance (EWV) (*58*), and Poincaré sphere internal volume (PSIV) (*59*, *60*). Geometrically, the independent analyzing SoPs define a polyhedron within the PS. Azzam *et al.* (*59*, *60*) proposed that the optimal set of analyzing SoPs is achieved when this inscribed polyhedron reaches its maximum volume.

We do not claim superiority of any particular optimization method; the PSIV approach is adopted here to demonstrate our analysis, but a similar methodology can be applied using other metrics. For illustrative purposes, we consider N=4 . The four independent SoPs serve as the vertices to construct a tetrahedron within the PS. The maximal volume is achieved when the configuration forms a regular tetrahedron, as illustrated in note S6. Previous demonstration shows that applying an intensity filter leads to an exclusion of certain SoPs, visually represented by a spherical cap. According to the geometric relationship, it is intuitive that the integrity of the regular tetrahedron configuration is preserved as long as the spherical cap’s cross section does not extend beyond the tetrahedron’s base. According to Eq. 33 in note S6, we have

$$9{\text{sin}}^{2}2\gamma =16+16\left(2F-1\right)+{\text{tan}}^{2}2\gamma {\left(2F-1\right)}^{2}$$ (2)

where γ represents the extinction ratio as defined in note S1 (Eq. 6), and *F* denotes the intensity threshold. Then, for a given intensity filter value *F*, we can deduce the boundary of diattenuation that maintains the optimal SoPs. Furthermore, the SN corresponding to the boundary diattenuation can also be calculated. Therefore, whenever the resulting SN remains within this limit, the system can operate optimally. When *E* increases and exceeds this boundary, the optimal configuration is compromised. However, it remains feasible to find four SoPs that maximize the volume of inscribed tetrahedron within the deficient PS. The related output SN provides an indication of the optimal performance this system can achieve under such a diattenuation, as detailed in note S7. To conclude, with the assistance of optical skyrmionic beams, the maximum tolerance of diattenuation for optimal Stokes vector measurement has been derived. The SN of the output beam serves as a metric to evaluate the system performance, providing a robust tool for practical application optimization.

## DISCUSSION

In summary, we harness a vectorial structured light field—Néel-type skyrmionic beam, as well as its SN, to evaluate quantitatively the correction ability of V-AO in the presence of nondepolarizing aberrations. We show that the limit of the AO system is not solely determined by the dynamic range and configuration of the AO devices but is significantly influenced by the inherent properties of the aberrations. Specifically, the intensity loss and polarization changes induced by diattenuation play a crucial role in defining the correctability boundaries of the system, taking the camera’s sensitivity into consideration.

We focused on precorrection of diattenuation aberration, where the correction ability is related to the intrinsic properties of diattenuation itself. Our results led to an interesting observation that, for a determined diattenuation aberration, certain precorrection of the SoPs can still be beneficial—i.e., if the desired SoP can be achieved in the output Stokes vector field, then this state can be produced through correction modules. Hence, for several important applications, such as polarization microscopy that suffers from diattenuation aberrations, V-AO may still take on a precorrection geometry and provide the required performance. Furthermore, by analyzing the properties of vectorial aberration, the maximum level of diattenuation aberration that can be corrected via AO to maintain the optimal performance of a Stokes-Mueller microscope was also derived.

There are intriguing areas to be further explored: (i) In real-world applications, such analysis provides a standard for determining appropriate correction strategies. For example, in scenarios where the aberration is beyond the AO correction boundary, current optimal solutions of the system may be unable to correct the aberrated field, and other compensation strategies, such as passive or hybrid compensation methods, may be needed; (ii) From our SN-assisted V-AO correction experiments, we found an interesting “duality”: The V-AO module, while targeting compensation of vectorial errors within its capacity, in effect, also dynamically optimizes polarimetric imaging performance via optimal channels under existing conditions. There remains intriguing scope for future exploration of such properties in both theoretical and experimental analyses; (iii) Besides Néel-type skyrmionic beams, other types of complex skyrmionic beams may also be used to optimally sense different diattenuation profiles. Hence, future studies can thus extend our methodology by exploring various optical skyrmion textures, investigating how each of them responds to distinct diattenuation patterns and/or optimizing the beam design accordingly. Such expansions will not only enhance the versatility of our technique but also provide more refined correction strategies for different aberrated scenarios. (iv) Furthermore, partially depolarized beams with diattenuation, as well as developments of novel AO devices, both deserve to be investigated to span across more real-world application scenarios. For example, this could extend to biomedical imaging, where thick tissues may depolarize light to varying degrees, or to remote sensing and telecommunications, where environmental factors can introduce partial depolarization and diattenuation. Different degrees of polarization will not only alter the mapping of SoPs but also affect the effect of the intensity filters, transforming its effect from a simple cap to a more complex shape. It will consequently also shift the optimal boundary for PSA. This necessitates a further analysis to comprehensively understand how variations in degree of polarization affect SoP loss, which will be crucial for better correction strategies and optimizing the system performance. (v) Although residual intensity issues remain to be resolved in current work, future applications that require more precise aberration correction, particularly in cases involving severe diattenuation, may benefit from incorporating other AO tools such as intensity adaptive optics (*22*), which could be used to enhance signal levels in affected regions, thereby facilitating more robust SoP recovery under challenging conditions.

Overall, our work paves the way for a previously unidentified usage of complex skyrmionic beams, as well as fundamental probing methodologies in next-generation V-AO. We believe that the potential applications of our work span from AO-enhanced sensing/imaging techniques to biomedical/material applications and represent a first step toward interdisciplinary research integrating optical skyrmions, AO, sample information analysis/characterization, as well as polarimetric measurements.

## METHODS

### Generation of optical skyrmion and measurement procedures

Néel-type optical skyrmionic beams were generated using a cascaded arrangement of three phase-only SLMs, each calibrated for accurate retardance modulation (*14*). A linearly polarized input beam, expanded to match the SLM aperture, passed through the three SLMs arranged in a folded 4f relay configuration to ensure pupil-plane conjugation. Quarter-wave plate (QWP) and polarizers were placed between the SLMs to define the polarization state incident on each modulator. The optical axes of the SLMs were oriented at 0° , 45° , and 0° , respectively, such that the system functioned as a composite elliptical retarder. This enabled structured phase modulation of orthogonal polarization components to generate the desired spatially varying Stokes vector field (*17*). The resulting polarization distribution formed a Néel-type skyrmion, with the SN determined by the programmed phase retardance profiles.

This system was modeled using Mueller matrices, with the cascaded SLMs transforming the input Stokes vector into a spatially varying output field. The generated beam was characterized via a Stokes polarimeter consisting of a QWP and a linear polarizer (*41*) to reconstruct the output Stokes vector field. The measurement steps are as follows: By rotating the QWP to four distinct angles, we captured intensity images of the entire beam using a charge-coupled device camera. Each pixel in these images corresponds to a specific point on the beam, allowing us to measure the SoP at each location—this is a widely used Stokes vector measurement procedure (*41*, *60*).

### Calculation of the SN

Subsequently, we calculated the corresponding SN of the output field. All measured Stokes parameters were normalized by $S_{0}$ to yield the SoP at each pixel. To reduce the influence of noise, the normalised Stokes vector field ( $S_{1}$ , $S_{2}$ , $S_{3}$ ) was smoothed using a Gaussian filter. Regions where the total intensity $S_{0}$ fell below the camera’s sensitivity threshold were excluded from further analysis to ensure reliability of the extracted polarization information. The SN was calculated from the spatial distribution of the normalized Stokes vectors using the topological charge integral as expressed in Eq. 1. Spatial derivatives were computed using central finite differences, and the integration was performed over the valid field of view after intensity-based masking. This calculation provides a global quantification of the polarization field, serving as the key metric for evaluating distortion due to diattenuation.

## Supplementary Materials

### This PDF file includes:

Notes S1 to S7 Figs. S1 to S6 References

## References

[1] T. Do, A. Hees, A. Ghez, G. D. Martinez, D. S. Chu, S. Jia, S. Sakai, J. R. Lu, A. K. Gautam, K. K. O’Neil, E. E. Becklin, M. R. Morris, K. Matthews, S. Nishiyama, R. Campbell, S. Chappell, Z. Chen, A. Ciurlo, A. Dehghanfar, E. Gallego-Cano, W. E. Kerzendorf, J. E. Lyke, S. Naoz, H. Saida, R. Schödel, M. Takahashi, Y. Takamori, G. Witzel, P. Wizinowich, Relativistic redshift of the star S0-2 orbiting the Galactic Center supermassive black hole. Science 365, 664–668 (2019). 31346138 10.1126/science.aav8137

[2] M. J. Booth, Adaptive optical microscopy: The ongoing quest for a perfect image. Light Sci. Appl. 3, e165 (2014).

[3] N. Ji, Adaptive optical fluorescence microscopy. Nat. Methods 14, 374–380 (2017). 28362438 10.1038/nmeth.4218

[4] H. Sun, J. Gersh-Range, N. J. Kasdin, “Modern wavefront control for space-based exoplanet coronagraph imaging,” in *2019 IEEE Aerospace Conference* (IEEE, 2019), pp. 1–10.

[5] Y. Wang, H. Xu, D. Li, R. Wang, C. Jin, X. Yin, S. Gao, Q. Mu, L. Xuan, Z. Cao, Performance analysis of an adaptive optics system for free-space optics communication through atmospheric turbulence. Sci. Rep. 8, 1124 (2018). 29348561 10.1038/s41598-018-19559-9 PMC5773697

[6] K. M. Hampson, R. Turcotte, D. T. Miller, K. Kurokawa, J. R. Males, N. Ji, M. J. Booth, Adaptive optics for high-resolution imaging. Nat. Rev. Methods Primers 1, 68 (2021). 35252878 10.1038/s43586-021-00066-7 PMC8892592

[7] P. S. Salter, M. J. Booth, Adaptive optics in laser processing. Light Sci. Appl. 8, 110 (2019). 31814967 10.1038/s41377-019-0215-1 PMC6884496

[8] R. Chipman, W. S. T. Lam, G. Young, *Polarized Light and Optical Systems* (CRC Press, 2018).

[9] C. He, J. Antonello, M. J. Booth, Vectorial adaptive optics. eLight 3, 23 (2023).

[10] C. He, H. He, J. Chang, B. Chen, H. Ma, M. J. Booth, Polarisation optics for biomedical and clinical applications: A review. Light Sci. Appl. 10, 194 (2021). 34552045 10.1038/s41377-021-00639-x PMC8458371

[11] H. He, R. Liao, N. Zeng, P. Li, Z. Chen, X. Liu, H. Ma, Mueller matrix polarimetry—An emerging new tool for characterizing the microstructural feature of complex biological specimen. J. Light. Technol. 37, 2534–2548 (2019).

[12] C. He, J. Chang, Q. Hu, J. Wang, J. Antonello, H. He, S. Liu, J. Lin, B. Dai, D. S. Elson, P. Xi, H. Ma, M. J. Booth, Complex vectorial optics through gradient index lens cascades. Nat. Commun. 10, 4264 (2019). 31537802 10.1038/s41467-019-12286-3 PMC6753074

[13] C. He, J. Chang, P. S. Salter, Y. Shen, B. Dai, P. Li, Y. Jin, S. C. Thodika, M. Li, T. Aziz, J. Wang, J. Antonello, Y. Dong, J. Qi, J. Lin, D. S. Elson, M. Zhang, H. He, H. Ma, M. J. Booth, Revealing complex optical phenomena through vectorial metrics. Adv. Photonics 4, 026001 (2022).

[14] C. He, B. Chen, Z. Song, Z. Zhao, Y. Ma, H. He, L. Luo, T. Marozsak, A. A. Wang, R. Xu, P. Huang, J. Li, X. Qiu, Y. Zhang, B. Sun, J. Cui, Y. Cai, Y. Zhang, A. Wang, M. Wang, P. Salter, J. A. J. Fells, B. Dai, S. Liu, L. Guo, Y. He, H. Ma, D. J. Royston, S. J. Elston, Q. Zhan, C. Qiu, S. M. Morris, M. J. Booth, A. Forbes, A reconfigurable arbitrary retarder array as complex structured matter. Nat. Commun. 16, 4902 (2025). 40425556 10.1038/s41467-025-59846-4 PMC12117100

[15] J. J. Gil, R. Ossikovski, *Polarized Light and the Mueller Matrix Approach* (CRC Press, 2022).

[16] Y. Ma, Z. Zhao, J. Cui, J. Wang, C. He, Vectorial adaptive optics for advanced imaging systems. J. Opt. 26, 065402 (2024).

[17] Y. Dai, C. He, J. Wang, R. Turcotte, L. Fish, M. Wincott, Q. Hu, M. J. Booth, Active compensation of extrinsic polarization errors using adaptive optics. Opt. Express 27, 35797–35810 (2019). 31878746 10.1364/OE.27.035797

[18] Q. Hu, Y. Dai, C. He, M. J. Booth, Arbitrary vectorial state conversion using liquid crystal spatial light modulators. Opt. Commun. 459, 125028 (2020).

[19] Q. Hu, C. He, M. J. Booth, Arbitrary complex retarders using a sequence of spatial light modulators as the basis for adaptive polarisation compensation. J. Opt. 23, 065602 (2021).

[20] C. He, M. J. Booth, “Enhancing polarisation imaging through novel polarimetry and adaptive optics,” in *Polarized Light and Optical Angular Momentum for Biomedical Diagnostics 2022*, J. C. Ramella-Roman, H. Ma, T. Novikova, D. S. Elson, I. A. Vitkin, Eds. (SPIE, 2022), vol. 11963, p. 1196302.

[21] C. He, M. J. Booth, “Vectorial adaptive optics: Correction of polarization and phase,” in *Imaging and Applied Optics Congress 2022* (paper OTh3B-4, Optica Publishing Group, 2022).

[22] Z. Zhao, Y. Ma, Z. Song, J. Antonello, J. Cui, B. Chen, J. Wang, B. Sun, H. He, L. Luo, J. A. J. Fells, S. J. Elston, M. J. Booth, S. M. Morris, C. He, Intensity adaptive optics. Light Sci. Appl. 14, 128 (2025). 40108147 10.1038/s41377-025-01779-0 PMC11923252

[23] T. H. R. Skyrme, A non-linear field theory. Proc. R. Soc. London Ser. A Math. Phys. Sci. 260, 127–138 (1961).

[24] A. Fert, N. Reyren, V. Cros, Magnetic skyrmions: Advances in physics and potential applications. Nat Rev. Mater. 2, 1–15 (2017).

[25] D. Foster, C. Kind, P. J. Ackerman, J.-S. B. Tai, M. R. Dennis, I. I. Smalyukh, Two-dimensional skyrmion bags in liquid crystals and ferromagnets. Nat. Phys. 15, 655–659 (2019).

[26] M. Król, H. Sigurdsson, K. Rechcińska, P. Oliwa, K. Tyszka, W. Bardyszewski, A. Opala, M. Matuszewski, P. Morawiak, R. Mazur, W. Piecek, P. Kula, P. G. Lagoudakis, B. Piętka, J. Szczytko, Observation of second-order meron polarization textures in optical microcavities. Optica 8, 255–261 (2021).

[27] S. Donati, L. Dominici, G. Dagvadorj, D. Ballarini, M. De Giorgi, A. Bramati, G. Gigli, Y. G. Rubo, M. H. Szymańska, D. Sanvitto, Twist of generalized skyrmions and spin vortices in a polariton superfluid. Proc. Natl. Acad. Sci. U.S.A. 113, 14926–14931 (2016). 27965393 10.1073/pnas.1610123114 PMC5206528

[28] C. He, Y. Shen, A. Forbes, Towards higher-dimensional structured light. Light Sci. Appl. 11, 205 (2022). 35790711 10.1038/s41377-022-00897-3 PMC9256673

[29] S. Tsesses, E. Ostrovsky, K. Cohen, B. Gjonaj, N. H. Lindner, G. Bartal, Optical skyrmion lattice in evanescent electromagnetic fields. Science 361, 993–996 (2018). 30026318 10.1126/science.aau0227

[30] L. Du, A. Yang, A. V. Zayats, X. Yuan, Deep-subwavelength features of photonic skyrmions in a confined electromagnetic field with orbital angular momentum. Nat. Phys. 15, 650–654 (2019).

[31] Y. Shen, C. He, Z. Song, B. Chen, H. He, Y. Ma, J. A. J. Fells, S. J. Elston, S. M. Morris, M. J. Booth, A. Forbes, Topologically controlled multiskyrmions in photonic gradient-index lenses. Phys. Rev. Appl. 21, 024025 (2024).

[32] Y. Shen, E. C. Martínez, C. Rosales-Guzmán, Generation of optical skyrmions with tunable topological textures. ACS Photonics 9, 296–303 (2022).

[33] I. Nape, K. Singh, A. Klug, W. Buono, C. Rosales-Guzman, A. McWilliam, S. Franke-Arnold, A. Kritzinger, P. Forbes, A. Dudley, Revealing the invariance of vectorial structured light in complex media. Nat. Photonics 16, 538–546 (2022).

[34] Y. Shen, Q. Zhang, P. Shi, L. Du, X. Yuan, A. V. Zayats, Optical skyrmions and other topological quasiparticles of light. Nat. Photonics 18, 15–25 (2024).

[35] A. A. Wang, Y. Ma, Y. Zhang, Z. Zhao, Y. Cai, X. Qiu, B. Dong, C. He, Unlocking new dimensions in photonic computing using optical skyrmions. arXiv:2407.16311 [physics.optics] (2024).

[36] S. Gao, F. C. Speirits, F. Castellucci, S. Franke-Arnold, S. M. Barnett, J. B. Götte, Paraxial skyrmionic beams. Phys. Rev. A 102, 053513 (2020).

[37] A. A. Wang, Z. Zhao, Y. Ma, Y. Cai, R. Zhang, X. Shang, Y. Zhang, J. Qin, Z.-K. Pong, T. Marozsák, B. Chen, H. He, L. Luo, M. J. Booth, S. J. Elston, S. M. Morris, C. He, Topological protection of optical skyrmions through complex media. Light Sci. Appl. 13, 314 (2024). 39572554 10.1038/s41377-024-01659-z PMC11582597

[38] B. Göbel, I. Mertig, O. A. Tretiakov, Beyond skyrmions: Review and perspectives of alternative magnetic quasiparticles. Phys. Rep. 895, 1–28 (2021).

[39] N. Nagaosa, Y. Tokura, Topological properties and dynamics of magnetic skyrmions. Nat. Nanotechnol. 8, 899–911 (2013). 24302027 10.1038/nnano.2013.243

[40] D. H. Goldstein, *Polarized Light* (CRC Press, 2017).

[41] R. M. A. Azzam, Stokes-vector and Mueller-matrix polarimetry. J. Opt. Soc. Am. A 33, 1396–1408 (2016). 10.1364/JOSAA.33.001396 27409699

[42] D. H. Goldstein, Mueller matrix dual-rotating retarder polarimeter. Appl. Opt. 31, 6676–6683 (1992). 20733896 10.1364/AO.31.006676

[43] P. Shukla, A. Awasthi, P. K. Pandey, A. Pradhan, “Discrimination of normal and dysplasia in cervix tissue by Mueller matrix analysis,” in *Biomedical Applications of Light Scattering II* (SPIE, 2008), vol. 6864, pp. 248–255.

[44] C. He, H. He, J. Chang, Y. Dong, S. Liu, N. Zeng, Y. He, H. Ma, Characterizing microstructures of cancerous tissues using multispectral transformed Mueller matrix polarization parameters. Biomed. Opt. Express 6, 2934–2945 (2015). 26309757 10.1364/BOE.6.002934 PMC4541521

[45] Y. Wang, H. He, J. Chang, C. He, S. Liu, M. Li, N. Zeng, J. Wu, H. Ma, Mueller matrix microscope: A quantitative tool to facilitate detections and fibrosis scorings of liver cirrhosis and cancer tissues. J. Biomed. Opt. 21, 071112 (2016). 10.1117/1.JBO.21.7.071112 27087003

[46] Y. Dong, J. Qi, H. He, C. He, S. Liu, J. Wu, D. S. Elson, H. Ma, Quantitatively characterizing the microstructural features of breast ductal carcinoma tissues in different progression stages by Mueller matrix microscope. Biomed. Opt. Express 8, 3643–3655 (2017). 28856041 10.1364/BOE.8.003643 PMC5560831

[47] J. Chang, H. He, Y. Wang, Y. Huang, X. Li, C. He, R. Liao, N. Zeng, S. Liu, H. Ma, Division of focal plane polarimeter-based 3× 4 Mueller matrix microscope: A potential tool for quick diagnosis of human carcinoma tissues. J. Biomed. Opt. 21, 056002 (2016). 10.1117/1.JBO.21.5.056002 27156716

[48] L. Deng, Z. Fan, B. Chen, H. Zhai, H. He, C. He, Y. Sun, Y. Wang, H. Ma, A dual-modality imaging method based on polarimetry and second harmonic generation for characterization and evaluation of skin tissue structures. Int. J. Mol. Sci. 24, 4206 (2023). 36835613 10.3390/ijms24044206 PMC9966533

[49] J. Qi, C. He, D. S. Elson, Real time complete Stokes polarimetric imager based on a linear polarizer array camera for tissue polarimetric imaging. Biomed. Opt. Express 8, 4933–4946 (2017). 29188092 10.1364/BOE.8.004933 PMC5695942

[50] H. He, C. He, J. Chang, D. Lv, J. Wu, C. Duan, Q. Zhou, N. Zeng, Y. He, H. Ma, Monitoring microstructural variations of fresh skeletal muscle tissues by Mueller matrix imaging. J. Biophotonics 10, 664–673 (2017). 27160958 10.1002/jbio.201600008

[51] C. He, H. He, X. Li, J. Chang, Y. Wang, S. Liu, N. Zeng, Y. He, H. Ma, Quantitatively differentiating microstructures of tissues by frequency distributions of Mueller matrix images. J. Biomed. Opt. 20, 105009 (2015). 26502227 10.1117/1.JBO.20.10.105009

[52] Z. Zhang, R. Hao, C. Shao, C. Mi, H. He, C. He, E. Du, S. Liu, J. Wu, H. Ma, Analysis and optimization of aberration induced by oblique incidence for in-vivo tissue polarimetry. Opt. Lett. 48, 6136–6139 (2023). 38039210 10.1364/OL.501365

[53] Z. Zhang, C. Shao, H. He, C. He, S. Liu, H. Ma, Analyzing the influence of oblique incidence on quantitative backscattering tissue polarimetry: A pilot *ex vivo* study. J. Biomed. Opt. 28, 102905 (2023). 37554626 10.1117/1.JBO.28.10.102905 PMC10406390

[54] J. Chang, H. He, C. He, Y. Wang, N. Zeng, R. Liao, H. Ma, Optimization of GRIN lens Stokes polarimeter. Appl. Opt. 54, 7424–7432 (2015). 26368781 10.1364/AO.54.007424

[55] C. Shao, B. Chen, H. He, C. He, Y. Shen, H. Zhai, H. Ma, Analyzing the influence of imaging resolution on polarization properties of scattering media obtained from Mueller matrix. Front. Chem. 10, 936255 (2022). 35903191 10.3389/fchem.2022.936255 PMC9315153

[56] C. He, J. Lin, J. Chang, J. Antonello, B. Dai, J. Wang, J. Cui, J. Qi, M. Wu, D. S. Elson, P. Xi, A. Forbes, M. J. Booth, Full Poincaré polarimetry enabled through physical inference. Optica 9, 1109–1114 (2022).

[57] V. V. Marenko, T. V. Molebnaya, Optimization of Stokes polarimeters employing a measurement of 4 intensities. Sov. J. Opt. Technol. 57, 452–455 (1990).

[58] D. S. Sabatke, M. R. Descour, E. L. Dereniak, W. C. Sweatt, S. A. Kemme, G. S. Phipps, Optimization of retardance for a complete Stokes polarimeter. Opt. Lett. 25, 802–804 (2000). 18064189 10.1364/ol.25.000802

[59] R. M. A. Azzam, I. M. Elminyawi, A. M. El-Saba, General analysis and optimization of the four-detector photopolarimeter. J. Opt. Soc. Am. A 5, 681–689 (1988).

[60] R. M. A. Azzam, Arrangement of four photodetectors for measuring the state of polarization of light. Opt. Lett. 10, 309–311 (1985). 19724430 10.1364/ol.10.000309

[61] R. A. Chipman, “Polarization Aberrations (Thin Films),” thesis, The University of Arizona, Tucson, AZ (1987).

[62] R. Ossikovski, Interpretation of nondepolarizing Mueller matrices based on singular-value decomposition. J. Opt. Soc. Am. A Opt. Image Sci. Vis. 25, 473–482 (2008). 18246182 10.1364/josaa.25.000473

[63] J. D. Muñoz-Bolaños, P. Rajaeipour, K. Kummer, M. Kress, C. Ataman, M. Ritsch-Marte, A. Jesacher, Confocal Raman microscopy with adaptive optics. ACS Photonics 12, 176–184 (2024). 39830861 10.1021/acsphotonics.4c01432 PMC11741161

[64] M. Veettikazhy, J. Nylk, F. Gasparoli, A. Escobet-Montalbán, A. K. Hansen, D. Marti, P. E. Andersen, K. Dholakia, Multi-photon attenuation-compensated light-sheet fluorescence microscopy. Sci. Rep. 10, 8090 (2020). 32415135 10.1038/s41598-020-64891-8 PMC7229186

