# Supplementary material for: Using optical skyrmions to assess vectorial adaptive optics capabilities in the presence of complex aberrations
Source: Sci Adv. 2025 Oct 3;11(40):eadv7904. doi: 10.1126/sciadv.adv7904 (PMC12494033; doi:10.1126/sciadv.adv7904)
Supplement: Supplementary file 1 — Notes S1 to S7 Figs. S1 to S6 References [file sciadv.adv7904_sm.pdf]

Science Advances
AAAS

Supplementary Materials for

Using optical skyrmions to assess vectorial adaptive optics capabilities in the presence of complex aberrations

Yifei Ma *et al.*

Corresponding author: Honghui He, he.honghui@sz.tsinghua.edu.cn; Lin Luo, luol@pku.edu.cn; Yonghong He, heyh@sz.tsinghua.edu.cn; Chao He, chao.he@eng.ox.ac.uk

Sci. Adv. **11**, eadv7904 (2025)
DOI: 10.1126/sciadv.adv7904

This PDF file includes:

Notes S1 to S7
Figs. S1 to S6
References

1 Mathematical representation of an arbitrary diattenuator

A diattenuator is an anisotropic attenuator that can non-uniformly attenuate the orthogonal components of a light field. The transmission ratios of the two orthogonal axes are denoted by p_x and p_y, then the orthogonal components of the light field are related by

$$E'_x = p_x E_x, 0 \leq p_x \leq 1 \tag{1}$$

$$E'_y = p_y E_y, 0 \leq p_y \leq 1 \tag{2}$$

where p_x and p_y are the attenuation coefficients and $0 \leq p_x, p_y \leq 1$. p_x (or p_y)=1 is for perfect transmission whereas p_x (or p_y)=0 is for complete attenuation. Normally, the normalised attenuation coefficients are defined in trigonometric function form:

$$p_x = \cos\gamma \tag{3}$$

$$p_y = \sin\gamma \tag{4}$$

$$p_x^2 + p_y^2 = 1. \tag{5}$$

For an arbitrary diattenuator, there are three parameters: extinction ratio (E), transmissive axis shape ($\mathbf{S}$) as well as orientation (θ). E is an intensity extinction ratio which is defined as the ratio between the attenuation coefficients of transmissive axis and orthogonal axes (normally we define $p_x \geq p_y$ to represent the attenuation coefficient of the transmissive axis),

$$E = \frac{p_x}{p_y} = \cot\gamma. \tag{6}$$

$\mathbf{S}$ represents the shape of the transmissive axis of the diattenuator which can be described with Jones vector as

$$\mathbf{S} = \begin{bmatrix} \cos\theta \\ \sin\theta e^{i\delta} \end{bmatrix}. \tag{7}$$

It has the exact same vectorial format as a light-wise Jones vector, but represents the object-wise property of the shape of the transmissive axis. Thus the Jones matrix of an arbitrary diattenuator can be expressed as:

$$\begin{aligned} \mathbf{J} &= \begin{bmatrix} \cos\theta & -\sin\theta e^{-i\delta} \\ \sin\theta e^{i\delta} & \cos\theta \end{bmatrix} \begin{bmatrix} \cos\gamma & 0 \\ 0 & \sin\gamma \end{bmatrix} \begin{bmatrix} \cos\theta & \sin\theta e^{-i\delta} \\ -\sin\theta e^{i\delta} & \cos\theta \end{bmatrix} \\ &= \begin{bmatrix} \cos\gamma\cos^2\theta + \sin\gamma\sin^2\theta & (\cos\gamma - \sin\gamma)\cos\theta\sin\theta e^{-i\delta} \\ (\cos\gamma - \sin\gamma)\cos\theta\sin\theta e^{i\delta} & \cos\gamma\sin^2\theta + \sin\gamma\cos^2\theta \end{bmatrix}. \end{aligned} \tag{8}$$

The Mueller matrix of an arbitrary diattenuator can be converted from a Jones matrix by:

$$\begin{aligned} \mathbf{M} &= \mathbf{A}(\mathbf{J} \otimes \mathbf{J}^*)\mathbf{A}^{-1} \\ &= \frac{1}{2}\begin{bmatrix} 1 & \mathbf{D}^{\mathrm{T}} \\ \mathbf{D} & \mathbf{m_D} \end{bmatrix} \end{aligned} \tag{9}$$

where $\otimes$ represents the kronecker product, $\mathbf{D}$ is the diattenuation vector, $\mathbf{m_D}$ represents the lower 3x3 sub-matrix, and $\mathbf{A}$ is

$$\mathbf{A} = \begin{bmatrix} 1 & 0 & 0 & 1 \\ 1 & 0 & 0 & -1 \\ 0 & 1 & 1 & 0 \\ 0 & i & -i & 0 \end{bmatrix}. \tag{10}$$

By replacing the eigenvector terms in $\mathbf{J}$ with Stokes vector notations:

$$S = \begin{bmatrix} s_0 \\ s_1 \\ s_2 \\ s_3 \end{bmatrix} = \begin{bmatrix} 1 \\ \cos 2\theta \\ \sin 2\theta\cos\delta \\ \sin 2\theta\sin\delta \end{bmatrix} = \begin{bmatrix} 1 \\ \mathbf{S} \end{bmatrix}, \tag{11}$$

we can get:

$$\mathbf{D} = \cos 2\gamma \begin{bmatrix} s_1 \\ s_2 \\ s_3 \end{bmatrix} = \cos 2\gamma\mathbf{S} \tag{12}$$

$$\mathbf{m_D} = \sin 2\gamma\mathbf{I}_{3\times 3} + (1 - \sin 2\gamma)\mathbf{S}\cdot\mathbf{S}^T. \tag{13}$$

2 Mathematical descriptions of incident and output fields after an arbitrary diattenuator

Using the skyrmion number (SN) as the evaluation metric enables direct assessment of vectorial adaptive optics (V-AO) correction capability without requiring explicit reconstruction of diattenuation parameters through matrix inversion or multiple measurements involving complex setups. As discussed in the Main article, the number of SoPs that remain detectable after passing through the diattenuation reflects the residual polarisation diversity, which is equivalent to V-AO system's remaining correction capability to enable effective aberration compensation. This directly benefits applications such as maintaining optimised SoPs required for imaging tasks in polarimetry.

The theoretical model presented in this section describes how an intensity threshold level F, defined by detector sensitivity, intersects with the Poincaré sphere representation of the SoP. When a skyrmionic beam passes through a diattenuator, intensity of certain output SoPs may be attenuated below the threshold. This results in a spherical cap being removed from the observable SoP domain. The radius of this cap is jointly determined by the threshold level F and the extinction ratio E, and its geometry is analytically described.

This framework enables the SN to serve as a global metric that quantitatively reflects the polarisation diversity retained in the system, providing a direct and interpretable assessment of V-AO correctability, with potential applications in fields such as biomedical imaging, astronomy, and communications.

For an arbitrary incident light,

$$S_{\text{in}} = \begin{bmatrix} S_{\text{in}_0} \\ S_{\text{in}_1} \\ S_{\text{in}_2} \\ S_{\text{in}_3} \end{bmatrix} = \begin{bmatrix} 1 \\ \mathbf{S}_{\text{in}} \end{bmatrix}. \tag{14}$$

Therefore the Stokes vector of the outgoing light can be expressed as:

$$S_{\text{out}} = \frac{1}{2}\begin{bmatrix} 1 & \mathbf{D}^{\text{T}} \\ \mathbf{D} & \mathbf{m_D} \end{bmatrix}\begin{bmatrix} 1 \\ \mathbf{S}_{\text{in}} \end{bmatrix} = \frac{1}{2}\begin{bmatrix} 1+\mathbf{D}^{\text{T}} * \mathbf{S}_{\text{in}} \\ \mathbf{D}+\mathbf{m_D} * \mathbf{S}_{\text{in}} \end{bmatrix} = \begin{bmatrix} I \\ \mathbf{S}_{\text{out}} \end{bmatrix}. \tag{15}$$

The intensity can be expressed as:

$$\begin{aligned} I &= S_{\text{out}_0} \\ &= \frac{1}{2}(1+\mathbf{D}^{\text{T}} * \mathbf{S}_{\text{in}}) \\ &= \frac{1}{2}(1+\cos 2\gamma \mathbf{S}^{T} * \mathbf{S}_{\text{in}}). \end{aligned} \tag{16}$$

And the polarisation vector can be expressed as:

$$\mathbf{S}_{\text{out}} = \frac{1}{2}(\mathbf{D}+\mathbf{m_D} * \mathbf{S}_{\text{in}}). \tag{17}$$

In Fig S1, we represent the simulated output Stokes fields of a *Néel-type* skyrmionic beam that are modulated by different diattenuators.

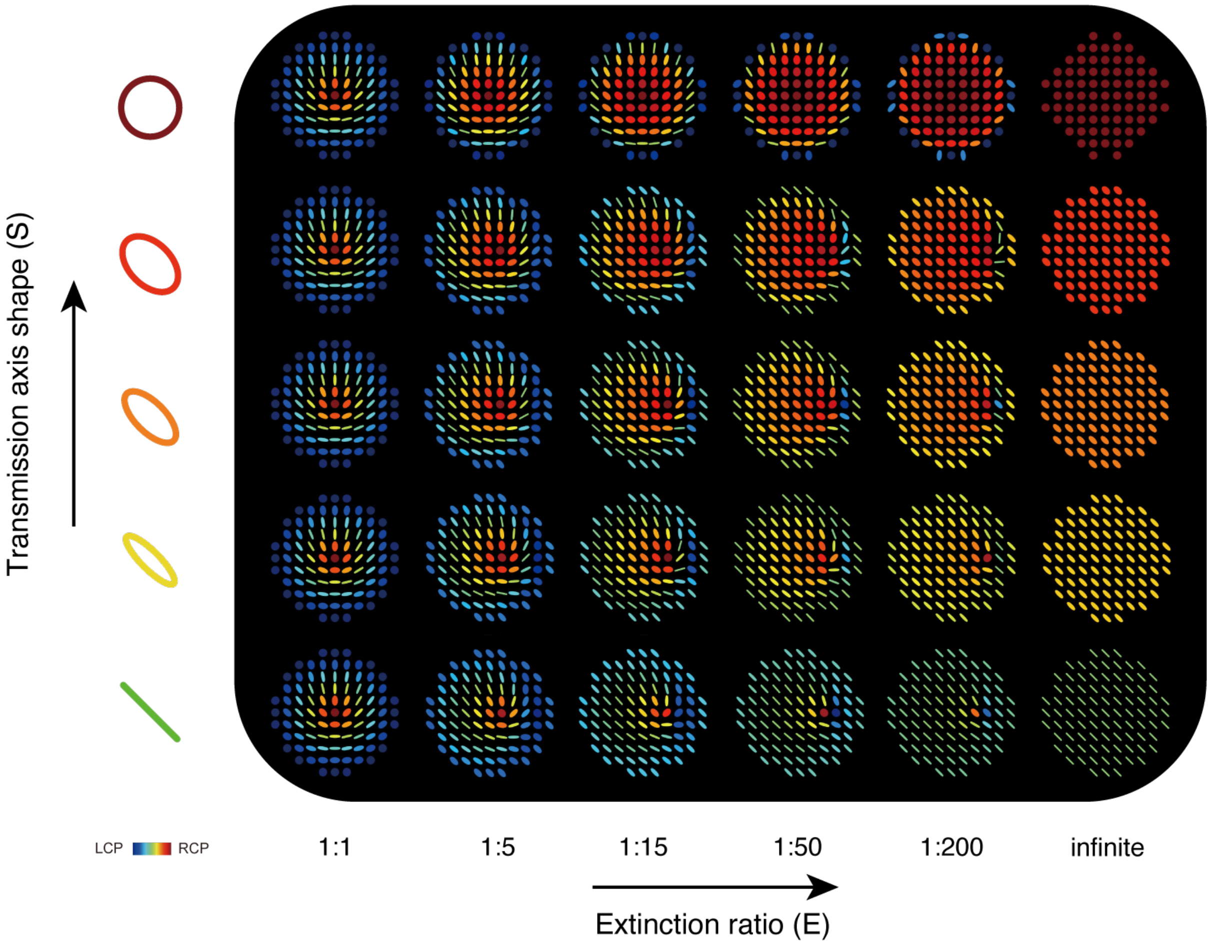

Figure S1: The output Stokes fields as a result of *Néel-type* skyrmionic beams being modulated by different diattenuators. The x axis of the figures represents different extinction rati (E) of the diattenuators, and the y axis represents different transmissive axis shapes $(\mathbf{S})$. The shaded region (dark grey region) represents the filtered out region when applying the intensity filter $F = 0.1$.

If we set a certain criterion to filter the output intensity, we can cut a certain region on the Poincaré sphere, assuming that the diattenuator has a non-ideal extinction ratio. Given that $I \leqslant F$ and assuming that $0 < \gamma < 45°$, we can derive:

$$\begin{aligned} I &\leqslant F \\ \frac{1}{2}(1 + \cos 2\gamma \mathbf{S}^T * \mathbf{S}_{\text{in}}) &\leqslant F \\ \mathbf{S}^T * \mathbf{S}_{\text{in}} &\leqslant \frac{2F-1}{\cos 2\gamma} \\ s_1 \cdot S_{\text{in}_1} + s_2 \cdot S_{\text{in}_2} + s_3 \cdot S_{\text{in}_3} &\leqslant \frac{2F-1}{\cos 2\gamma}. \end{aligned} \tag{18}$$

As $\mathbf{S}$ and $\mathbf{S}_{\text{in}}$ are from the normalised Stokes vector, thus $s_1^2 + s_2^2 + s_3^2 = 1$ and $\mathbf{S}_{\text{in}_1}^2 + \mathbf{S}_{\text{in}_2}^2 + \mathbf{S}_{\text{in}_3}^2 = 1$. So,

$$\begin{aligned} (s_1 + S_{\text{in}_1})^2 + (s_2 + S_{\text{in}_2})^2 + (s_3 + S_{\text{in}_3})^2 &\leqslant \frac{4F-2}{\cos 2\gamma} + 2 \\ ((-s_1) - S_{\text{in}_1})^2 + ((-s_2) - S_{\text{in}_2})^2 + ((-s_3) - S_{\text{in}_3})^2 &\leqslant \frac{4F-2}{\cos 2\gamma} + 2. \end{aligned} \tag{19}$$

It turns out that the incident light which will be filtered out after passing through the arbitrary diattenuator will cover a spherical cap on the Poincaré sphere which is cut by a plane perpendicular to the axis $[-s_1, -s_2, -s_3]$. And the max distance between the filtered Stokes vector and the axis vector is defined as:

$$\mathcal{D} = \sqrt{\frac{4F-2}{\cos 2\gamma} + 2} \tag{20}$$

where it implicitly defines:

$$\begin{aligned} 2 \geqslant \frac{4F-2}{\cos 2\gamma} + 2 \geqslant 0 \\ \frac{1}{2} \geqslant F \geqslant \frac{1-\cos 2\gamma}{2} \in (0, \frac{1}{2}). \end{aligned} \tag{21}$$

So we can define the boundary condition for the filter as:

$$\mathbf{S}^{\mathrm{T}} * \mathbf{S}_{\mathrm{in}} = s_1 \cdot S_{\mathrm{in}_1} + s_2 \cdot S_{\mathrm{in}_2} + s_3 \cdot S_{\mathrm{in}_3} = \frac{2F-1}{\cos 2\gamma} = \mathcal{X} \in (-1, 1). \tag{22}$$

Then according to the geometric relationship, the radius of the corresponding latitude circle can be expressed as:

$$\mathcal{R}_{\mathrm{in}} = \mathcal{D}\sqrt{1 - \left(\frac{\mathcal{D}}{2}\right)^2} = \sqrt{1-\mathcal{X}^2}. \tag{23}$$

It can also be proved that, for a given intensity filter value F, the output light that is filtered out will cover a spherical cap on the Poincaré sphere, which is cut by a plane perpendicular to the axis $[-s_1, -s_2, -s_3]$. This filtering not only defines the polarisation region retained after diattenuation but also sets the domain over which subsequent system metrics, such as SN, are evaluated. This can also be demonstrated by calculating the distance between the polarisation vector (normalised Stokes vector) and eigenvector $-\mathbf{S}$, which is:

$$\begin{aligned} \mathcal{D}^2 = \|\hat{\mathbf{S}}_{\mathrm{out}} + \mathbf{S}\|^2 &= \left\| \frac{\cos 2\gamma \mathbf{S} + \mathbf{m_D} * \mathbf{S}_{\mathrm{in}}}{1 + \cos 2\gamma \mathbf{S}^{\mathrm{T}} * \mathbf{S}_{\mathrm{in}}} + \mathbf{S} \right\|^2 \\ &= \left\| \frac{(1+\cos 2\gamma)\mathbf{S} + (\mathbf{m_D} + \cos 2\gamma \mathbf{S}\mathbf{S}^{\mathrm{T}}) * \mathbf{S}_{\mathrm{in}}}{1 + \cos 2\gamma \mathbf{S}^{\mathrm{T}} * \mathbf{S}_{\mathrm{in}}} \right\|^2 \\ &= \left\| \frac{(1+\cos 2\gamma)\mathbf{S} + \mathbf{m_D}' * \mathbf{S}_{\mathrm{in}}}{1 + \cos 2\gamma \mathbf{S}^{\mathrm{T}} * \mathbf{S}_{\mathrm{in}}} \right\|^2 \end{aligned} \tag{24}$$

where $\hat{\mathbf{S}}_{\mathrm{out}}$ is the normalised vector of $\mathbf{S}_{\mathrm{out}}$ and we define $\mathbf{m_D}'$:

$$\begin{aligned} \mathbf{m_D}' &= \mathbf{m_D} + \cos 2\gamma \mathbf{S}\mathbf{S}^{\mathrm{T}} \\ &= \begin{bmatrix} \sin 2\gamma + (1-\sin 2\gamma + \cos 2\gamma)s_1^2 & (1-\sin 2\gamma + \cos 2\gamma)s_1 s_2 & (1-\sin 2\gamma + \cos 2\gamma)s_1 s_3 \\ (1-\sin 2\gamma + \cos 2\gamma)s_1 s_2 & \sin 2\gamma + (1-\sin 2\gamma + \cos 2\gamma)s_2^2 & (1-\sin 2\gamma + \cos 2\gamma)s_2 s_3 \\ (1-\sin 2\gamma + \cos 2\gamma)s_1 s_3 & (1-\sin 2\gamma + \cos 2\gamma)s_2 s_3 & \sin 2\gamma + (1-\sin 2\gamma + \cos 2\gamma)s_3^2 \end{bmatrix} \\ &= \begin{bmatrix} m'_{D11} & m'_{D12} & m'_{D13} \\ m'_{D21} & m'_{D22} & m'_{D23} \\ m'_{D31} & m'_{D32} & m'_{D33} \end{bmatrix}. \end{aligned} \tag{25}$$

We know that:

$$\begin{aligned} \mathcal{X} &= s_1 \cdot S_{\mathrm{in}_1} + s_2 \cdot S_{\mathrm{in}_2} + s_3 \cdot S_{\mathrm{in}_3} \\ \mathcal{X}^2 &= s_1^2 S_{\mathrm{in}_1}^2 + s_2^2 S_{\mathrm{in}_2}^2 + s_3^2 S_{\mathrm{in}_3}^2 + 2 s_1 S_{\mathrm{in}_1} s_2 S_{\mathrm{in}_2} + 2 s_1 S_{\mathrm{in}_1} s_3 S_{\mathrm{in}_3} + 2 s_2 S_{\mathrm{in}_2} s_3 S_{\mathrm{in}_3}. \end{aligned} \tag{26}$$

Then it can be calculated that,

$$\begin{aligned} (1+\cos 2\gamma \mathcal{X})^2 \mathcal{D}^2 &= 2(1+\cos 2\gamma) + 2(1+\cos 2\gamma)^2 \mathcal{X} + 2\cos 2\gamma(1+\cos 2\gamma)\mathcal{X}^2 \\ &= 2(1+\cos 2\gamma)\left[1 + (1+\cos 2\gamma)\mathcal{X} + \cos 2\gamma \mathcal{X}^2\right] \\ &= 2(1+\cos 2\gamma)(1+\mathcal{X})(1+\cos 2\gamma \mathcal{X}). \end{aligned} \tag{27}$$

Thus,

$$\mathcal{D}^2 = \frac{2(1+\cos 2\gamma)(1+\mathcal{X})}{1+\cos 2\gamma \mathcal{X}}. \tag{28}$$

The radius of the corresponding latitude circle in the output field can be expressed as:

$$\mathcal{R}_{\mathrm{out}} = \mathcal{D}\sqrt{1 - \left(\frac{\mathcal{D}}{2}\right)^2} = \frac{\sin 2\gamma}{1+\cos 2\gamma \mathcal{X}}\sqrt{1-\mathcal{X}^2}. \tag{29}$$

3 SLM calibration process

Each SLM was calibrated to achieve precise and spatially uniform phase modulation across the entire 0 to 2π range. The SLM was placed between two linear polarisers, both set at 45° relative to the nematic liquid crystal (LC) director axis, enabling modulation of the transmitted intensity as a function of the voltage-induced phase retardation. Although the calibration involved measuring output intensity, the objective was to retrieve the corresponding phase shift introduced by each pixel.

The transmittance of the SLM system follows the relation:

$$T = \sin^2(2\chi)\sin^2\left(\frac{\pi\delta nd}{\lambda}\right) \tag{30}$$

where $\chi = 45°$, δn is the birefringence of the LC layer, d is the thickness of LC layer, and λ is the operating wavelength. This relation maps the change in voltage (applied via 8-bit grayscale values F_i) to a phase shift via its effect on birefringence. By recording the intensity transmitted through the SLM for flat grayscale patterns $F_i \in [0, 255]$, the phase response curve for each pixel was reconstructed from the measured sinusoidal intensity modulation using an inverse fitting procedure.

To ensure spatial accuracy, interpolation was used to match each SLM pixel with the corresponding camera pixel, eliminating resolution mismatches. For each pixel n, the intensity response $P_{n,i}$ was mapped across all grayscale levels, and the corresponding phase $\varphi_{n,i}$ was derived. The monotonic regions within the modulation curve were identified to avoid ambiguities in the inverse mapping. A pixel-specific look-up table (LUT) was then constructed to link grayscale input to calibrated phase values within the reliable modulation range.

During experimental operation, this LUT allowed each desired phase profile to be accurately rendered by selecting the appropriate grayscale values across the SLM. The laser intensity and camera exposure were optimised and then fixed throughout the calibration to maximise signal-to-noise ratio. This phase-based calibration ensured precise and repeatable encoding of structured phase patterns such as those used for generating skyrmion beams.

We note the cascaded SLMs may reduce the overall intensity level for imaging. The 2 mW He-Ne laser used in our setup delivered sufficient intensity for V-AO-enhanced conventional Stokes and Mueller matrix polarimetry experiments under a simple uniform filtering value. We also note that for other potential uses of V-AO (e.g., polarisation super-resolution microscopy), the loss of intensity through SLMs may become more critical – especially for polarisation optimization. However, in such cases, similar methodologies can be applied using higher-efficiency AO devices, more flexible light sources, or more sophisticated algorithms to meet the specific performance requirements [6, 63, 64].

4 Characterisation of diattenuation

We experimentally characterised the diattenuation properties using conventional Mueller matrix and Stokes polarimetry [41, 42]. This includes characterisation of the extinction ratio E, the shape of transmissive axis S, and the axis orientation θ. As shown in Fig.S2, representative measurements are presented for six diattenuation configurations (A-F), corresponding to extinction ratios $E = 1$, 1.65, 2.67, 3.36, 4.16, and 724.79, Configurations A to E were equivalently obtained using either single or cascaded weak diattenuators (i.e., beam splitters here), while the configuration F employed commercial polarisers to simulate strong diattenuators, theoretically approaching infinite values. As E increases, the output SoPs converge towards the transmissive axis of the diattenuator.

For each diattenuation configuration shown in Fig. S2, we first measured the corresponding Mueller matrix of the sample using a standard polarimetric setup [42]; these results are presented in the first row of the figure. To illustrate the effect of each diattenuation, three representative input SoPs – horizontal linear, right-circular, and +45° linear – were applied, and the resulting output fields were recorded using Stokes polarimetry and displayed in the remaining three rows. The measured results serve as a quantitative basis that supports the skyrmion-based method, which achieves direct assessment of system correctability without requiring multiple input SoPs or full Mueller matrix reconstruction.

These experiments were conducted using spatially uniform samples to ensure control and interpretability of the results (as mentioned before), thereby providing a reliable basis for defining the correction boundary. Once the underlying correction limits are known, the system's pixelated control architecture can be applied effectively to achieve aberration correction.

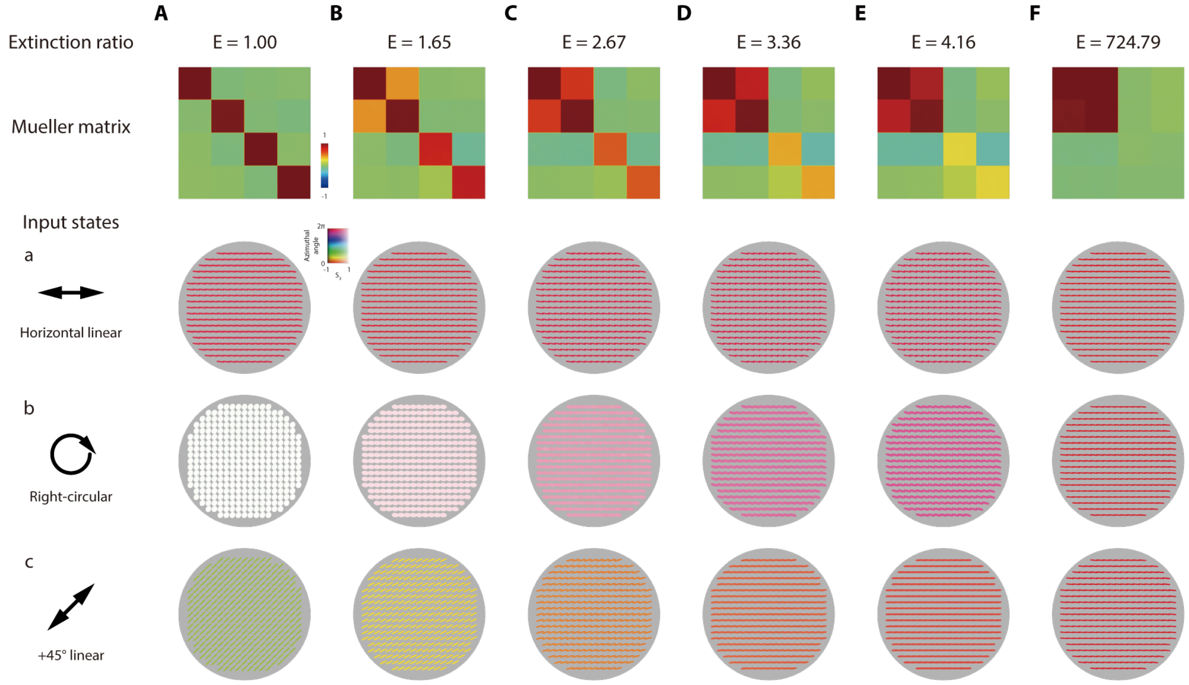

Figure S2: Experimental diattenuation characterisation via Mueller and Stokes polarimetry. Six representative diattenuation cases (A–F) with increasing extinction ratios ($E = 1$, 1.65, 2.67, 3.36, 4.16, and 724.79) are shown. For each configuration, the top row presents the measured Mueller matrix, then followed by the output Stokes fields for three input states – (a) horizontal linear, (b) right-circular, and (c) +45° linear – in the rest of the rows. As E increases, the output fields gradually converge towards the diattenuation's transmissive axis, revealing the stronger attenuation against the orthogonal component.

The simulation approach uses a look-up table (LUT), constructed by simulating the Jones matrices of diattenuators across a range of the key parameters, including extinction ratio, axis orientation, and shape. These matrices are used to simulate the corresponding diattenuation effects under various configurations, and the results are recorded into the LUT. To find the matched simulation results, the experimental diattenuation parameters for each sample, measured using Mueller matrix polarimetry [42], are mapped to the closest entries in the LUT.

The results demonstrate that our LUT-based simulation framework can reveal physical characteristics of the samples and provide a solid basis for exploring the potential of further skyrmion-based probing approaches as a cost-effective method for diattenuation analysis.

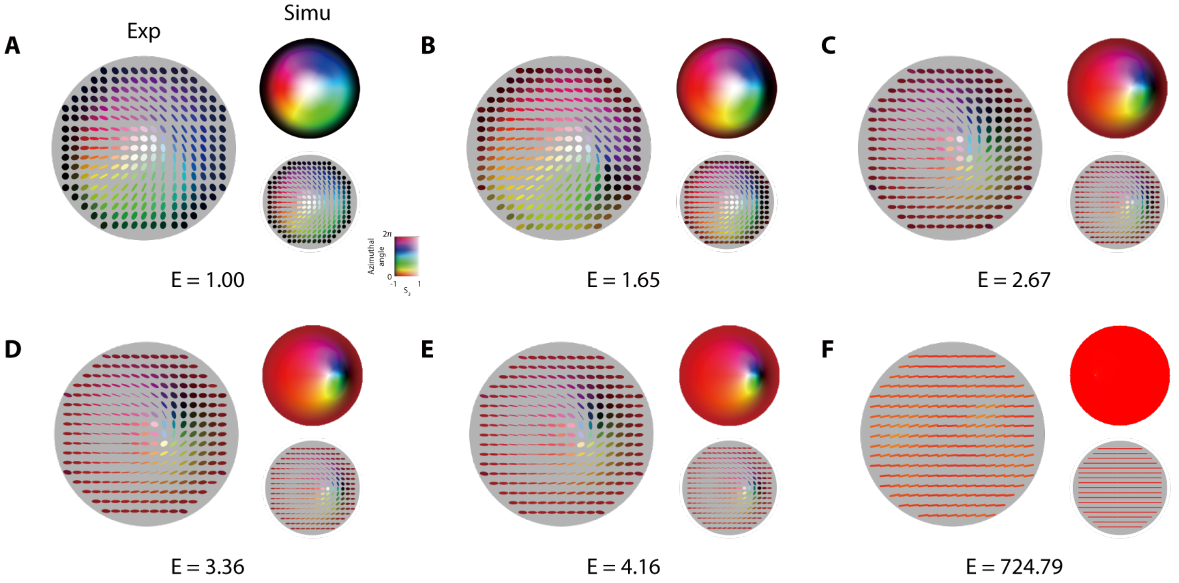

Figure S3: Experimental and simulated Stokes fields with corresponding skyrmionic structures under varying diattenuations. Stokes fields measured experimentally after propagation through diattenuators with extinction ratios (E) of (**A**) E=1, (**B**) E=1.65, (**C**) E=2.67, (**D**) E=3.36, (**E**) E=4.16, and (**F**) E=724.79 are displayed to the left. For each case, the corresponding simulated Stokes field and skyrmionic structure are presented to the right.

Fig. S3 shows the simulated and experimentally measured Stokes fields, together with the simulated skyrmionic fields under six different diattenuation conditions ($E = 1$, 1.65, 2.67, 3.36, 4.16, and 724.79). The experimental results align well with the simulations, but some minor discrepancies still remain. The residual errors from our experimental results (especially polarisation) can generally be accumulated from four key sources [9, 10, 16, 21, 43]: 1) light-generation imperfections. Manufacturing tolerances in the laser and residual calibration error in the cascaded SLMs (e.g., voltage-to-phase non-linearity) slightly distort the intended input SoP distribution. 2) sample and optical-path imperfection. Finite surface quality and unavoidable alignment errors (e.g. tip/tilt, decentering) in the diattenuation samples and relay optics introduce weak, unmodelled aberrations. 3) noise in measurements. Shot noise and read-out noise from the standard-sensitivity CMOS camera introduce random error, particularly in low-intensity regions surviving the diattenuation filter. 4) AO system calibration error. Residual errors in the phase-retardance look-up tables propagate into the pre-correction patterns, introducing small mismatch.

While each contribution is individually minor, their combined effect accounts for the deviations observed between experimental and simulated results. We note that these differences remain within the expected tolerance for a proof-of-concept study and also point toward directions for future engineering development and enhancements.

5 Quantitative analysis of V-AO correction

To further demonstrate the effectiveness of V-AO correction under diattenuation aberrations, we present an additional quantitative comparison in Fig. S4. For both the archaeological and biomedical samples, the three Stokes vector components S_1, S_2, S_3 are visualised under three conditions: 1) ground truth; 2) before V-AO correction under a moderate diattenuation ($E = 1.65$); 3) after V-AO correction. For each component, the value profile along the selected cross-section is presented. The close match between the V-AO corrected and ground-truth curves confirms the effectiveness of V-AO in restoring the polarisation field in the presence of diattenuations aberrations, enabling robust measurements in complex environments.

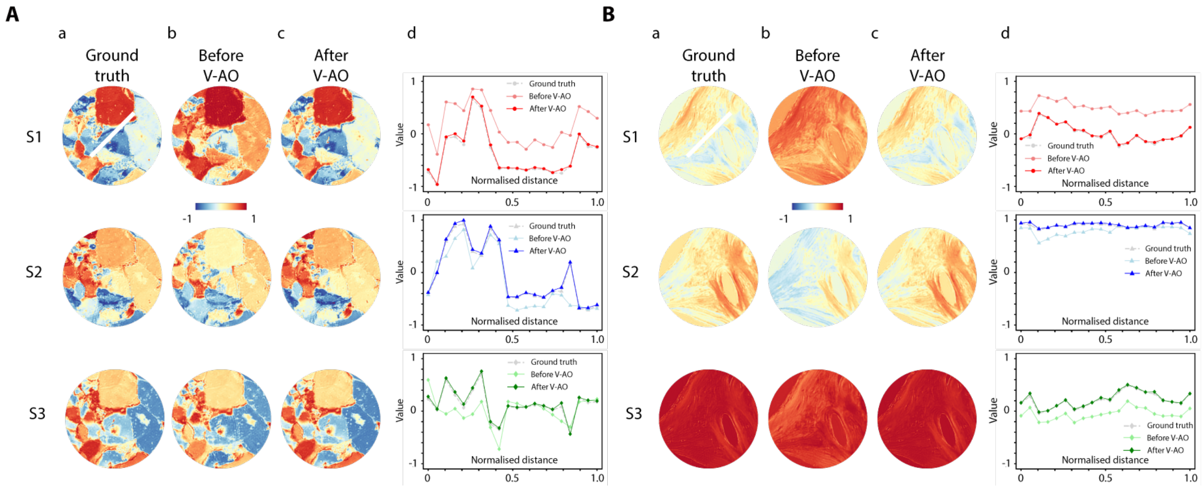

Figure S4: Stokes parameter fields evaluation of V-AO correction performance. Stokes parameter fields and numerical analyses of (A) archaeological sample and (B) biomedical sample. For each sample, the Stokes parameters for (a) ground truth, (b) before V-AO correction under a moderate diattenuation ($E = 1.65$) and (c) after V-AO correction are illustrated; (d) evaluates the V-AO correction performance by plotting the values of each Stokes parameter along a selected cross-section.

6 Maximum tolerance of diattenuation aberrations for optimal Stokes vector measurement

The maximum tolerance of diattenuation is mathematically determined here using geometric arguments. Consider a sphere with radius r and an inscribed regular tetrahedron with edge length a, where $AO = r$, $AC = a$. From the geometry, we have $r = \frac{\sqrt{6}}{4}a, \quad OH = \frac{\sqrt{6}}{12}a, \quad \frac{OH}{OC} = \frac{1}{3}$.

The surface area enclosed by the cone with vertices O, B, C and D can be calculated through surface integral:

$$S = \int_{\varphi=0}^{\varphi'} \int_{\theta=0}^{\theta=2\pi} R^2 \sin^2 \varphi d\varphi d\theta = 2\pi R^2 \tag{31}$$

where $\cos\varphi' = \frac{1}{3}$, the maximal reduced superficial area can be quantified as $\frac{4}{3}\pi R^2$.

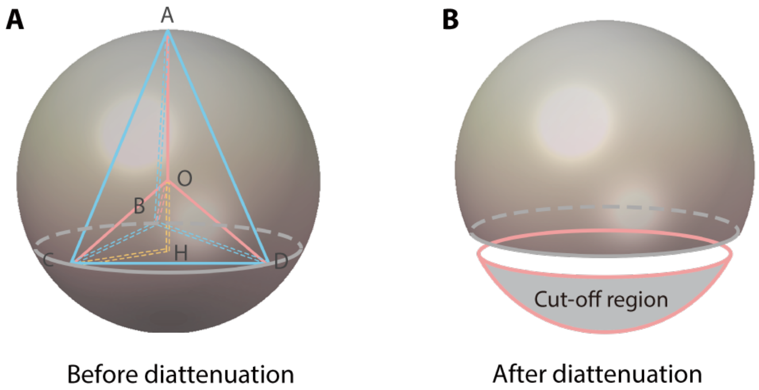

Figure S5: Geometrical visualisation of maximum tolerance of diattenuation aberration for optimal Stokes vector measurement. (A) Before diattenuation, an inscribed regular tetrahedron in PS defined by four optimal SoPs at vertices A, B, C and D. Point O represents the central point of the sphere, point H denotes the central point of the tetrahedron's base. (B) After diattenuation, the filtered output field covers a spherical cap (the grey region) in the PS surface, the radius of its cross section is related to the intensity filter value F.

We reach the boundary when $R = \frac{\sqrt{8}}{3}r$, according to Eq. (29) we have:

$$9\sin^2 2\gamma = 16 + 16(2F - 1) + \tan^2 2\gamma(2F - 1)^2 \tag{32}$$

where γ represents extinction ratio as defined in Eq. (6), F denotes the intensity threshold.

7 SN assisted V-AO for Stokes polarimetric imaging under different diattenuation cases

To demonstrate how SN serves as a practical indicator for guiding V-AO correction for optimal Stokes polarimetric imaging, five representative cases are illustrated in Fig. S6: two within the boundary (A and B), one on the boundary (C), and two beyond it (D and E). For each case, both the residual region on the Poincaré sphere and the largest inscribable tetrahedron (representing optimised measurement states) are shown.

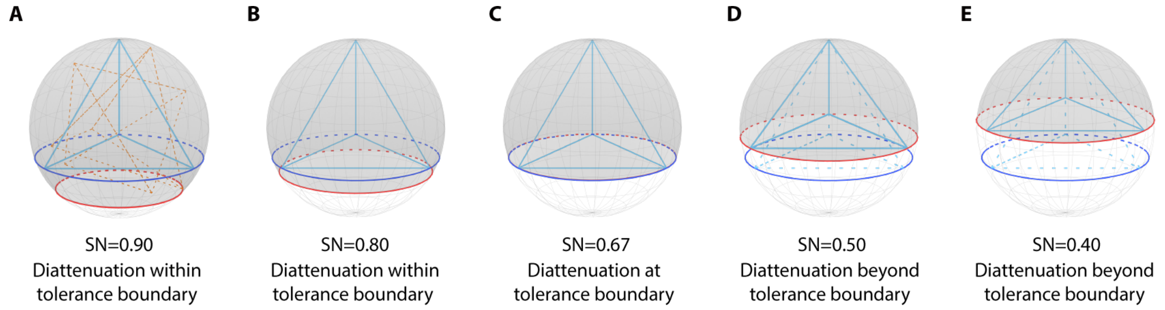

Figure S6: Optimal analysing SoPs for Stokes vector measurement under different diattenuation cases. (A)-(B): diattenuation values within the tolerance boundary, the dashed orange outline shows rotated yet equivalent optimal tetrahedrons; (C): diattenuation value at the tolerance boundary; (D)-(E): diattenuation values beyond the tolerance boundary. Both the remaining portion of the sphere and the largest tetrahedron that can be inscribed in each case are illustrated.

For diattenuation levels within the tolerance boundary (A-C), the accessible surface on the Poincaré sphere is sufficiently large to accommodate a full regular tetrahedron. Under these conditions, the original four analysing SoPs – and therefore the existing SLM phase settings – already support optimal measurement. For diattenuation levels beyond the tolerance boundary (D and E), if we aim to optimise the measurement states via V-AO in analysing arm (by controlling the SLM parameters), then the four analysing SoP points of the SN-linked tetrahedron (representing four optimised states) need to be realised by the SLMs' retardance settings (methods can be similar to Ref [17]). In this way, SN provides a clear and quantitative link with V-AO system, offering a practical metric for system optimisation – through SLM parameter control – even beyond ideal conditions.

It is also worth noting that in all cases a family of equivalent optimal SoPs exists, because the tetrahedron can be freely rotated about the sphere (as shown in Fig. S6A). This rotational freedom gives the control algorithm flexibility to choose whichever set of four SoPs is most convenient for implementation.

While this section demonstrates the use of SN to guide V-AO correction towards optimal Stokes polarimetric imaging, the underlying principle holds potential for broader applications in more complex measurement scenarios. For instance, extending the current methodology from uniform to spatially variant diattenuation profiles could enable corrections in increasingly realistic environments. Furthermore, systematically investigating how the spatial placement of vectorial aberrations impacts both polarisation generation and detection, as well as exploring the potential of SN as a versatile indicator for system robustness, calibration, or adaptive control, presents intriguing avenues for future research.

REFERENCES AND NOTES

1. T. Do, A. Hees, A. Ghez, G. D. Martinez, D. S. Chu, S. Jia, S. Sakai, J. R. Lu, A. K. Gautam, K. K. O'Neil, E. E. Becklin, M. R. Morris, K. Matthews, S. Nishiyama, R. Campbell, S. Chappell, Z. Chen, A. Ciurlo, A. Dehghanfar, E. Gallego-Cano, W. E. Kerzendorf, J. E. Lyke, S. Naoz, H. Saida, R. Schödel, M. Takahashi, Y. Takamori, G. Witzel, P. Wizinowich, Relativistic redshift of the star S0-2 orbiting the Galactic Center supermassive black hole. *Science* **365**, 664–668 (2019).

2. M. J. Booth, Adaptive optical microscopy: The ongoing quest for a perfect image. *Light Sci. Appl.* **3**, e165 (2014).

3. N. Ji, Adaptive optical fluorescence microscopy. *Nat. Methods* **14**, 374–380 (2017).

4. H. Sun, J. Gersh-Range, N. J. Kasdin, "Modern wavefront control for space-based exoplanet coronagraph imaging," in *2019 IEEE Aerospace Conference* (IEEE, 2019), pp. 1–10.

5. Y. Wang, H. Xu, D. Li, R. Wang, C. Jin, X. Yin, S. Gao, Q. Mu, L. Xuan, Z. Cao, Performance analysis of an adaptive optics system for free-space optics communication through atmospheric turbulence. *Sci. Rep.* **8**, 1124 (2018).

6. K. M. Hampson, R. Turcotte, D. T. Miller, K. Kurokawa, J. R. Males, N. Ji, M. J. Booth, Adaptive optics for high-resolution imaging. *Nat. Rev. Methods Primers* **1**, 68 (2021).

7. P. S. Salter, M. J. Booth, Adaptive optics in laser processing. *Light Sci. Appl.* **8**, 110 (2019).

8. R. Chipman, W. S. T. Lam, G. Young, *Polarized Light and Optical Systems* (CRC Press, 2018).

9. C. He, J. Antonello, M. J. Booth, Vectorial adaptive optics. *eLight* **3**, 23 (2023).

10. C. He, H. He, J. Chang, B. Chen, H. Ma, M. J. Booth, Polarisation optics for biomedical and clinical applications: A review. *Light Sci. Appl.* **10**, 194 (2021).

11. H. He, R. Liao, N. Zeng, P. Li, Z. Chen, X. Liu, H. Ma, Mueller matrix polarimetry—An emerging new tool for characterizing the microstructural feature of complex biological specimen. *J. Light. Technol.* **37**, 2534–2548 (2019).

12. C. He, J. Chang, Q. Hu, J. Wang, J. Antonello, H. He, S. Liu, J. Lin, B. Dai, D. S. Elson, P. Xi, H. Ma, M. J. Booth, Complex vectorial optics through gradient index lens cascades. *Nat. Commun.* **10**, 4264 (2019).

13. C. He, J. Chang, P. S. Salter, Y. Shen, B. Dai, P. Li, Y. Jin, S. C. Thodika, M. Li, T. Aziz, J. Wang, J. Antonello, Y. Dong, J. Qi, J. Lin, D. S. Elson, M. Zhang, H. He, H. Ma, M. J. Booth, Revealing complex optical phenomena through vectorial metrics. *Adv. Photonics* **4**, 026001 (2022).

14. C. He, B. Chen, Z. Song, Z. Zhao, Y. Ma, H. He, L. Luo, T. Marozsak, A. A. Wang, R. Xu, P. Huang, J. Li, X. Qiu, Y. Zhang, B. Sun, J. Cui, Y. Cai, Y. Zhang, A. Wang, M. Wang, P. Salter, J. A. J. Fells, B. Dai, S. Liu, L. Guo, Y. He, H. Ma, D. J. Royston, S. J. Elston, Q. Zhan, C. Qiu, S. M. Morris, M. J. Booth, A. Forbes, A reconfigurable arbitrary retarder array as complex structured matter. *Nat. Commun.* **16**, 4902 (2025).

15. J. J. Gil, R. Ossikovski, *Polarized Light and the Mueller Matrix Approach* (CRC Press, 2022).

16. Y. Ma, Z. Zhao, J. Cui, J. Wang, C. He, Vectorial adaptive optics for advanced imaging systems. *J. Opt.* **26**, 065402 (2024).

17. Y. Dai, C. He, J. Wang, R. Turcotte, L. Fish, M. Wincott, Q. Hu, M. J. Booth, Active compensation of extrinsic polarization errors using adaptive optics. *Opt. Express* **27**, 35797–35810 (2019).

18. Q. Hu, Y. Dai, C. He, M. J. Booth, Arbitrary vectorial state conversion using liquid crystal spatial light modulators. *Opt. Commun.* **459**, 125028 (2020).

19. Q. Hu, C. He, M. J. Booth, Arbitrary complex retarders using a sequence of spatial light modulators as the basis for adaptive polarisation compensation. *J. Opt.* **23**, 065602 (2021).

20. C. He, M. J. Booth, "Enhancing polarisation imaging through novel polarimetry and adaptive optics," in *Polarized Light and Optical Angular Momentum for Biomedical Diagnostics 2022*, J. C. Ramella-Roman, H. Ma, T. Novikova, D. S. Elson, I. A. Vitkin, Eds. (SPIE, 2022), vol. 11963, p. 1196302.

21. C. He, M. J. Booth, "Vectorial adaptive optics: Correction of polarization and phase," in *Imaging and Applied Optics Congress 2022* (paper OTh3B-4, Optica Publishing Group, 2022).

22. Z. Zhao, Y. Ma, Z. Song, J. Antonello, J. Cui, B. Chen, J. Wang, B. Sun, H. He, L. Luo, J. A. J. Fells, S. J. Elston, M. J. Booth, S. M. Morris, C. He, Intensity adaptive optics. *Light Sci. Appl.* **14**, 128 (2025).

23. T. H. R. Skyrme, A non-linear field theory. *Proc. R. Soc. London Ser. A Math. Phys. Sci.* **260**, 127–138 (1961).

24. A. Fert, N. Reyren, V. Cros, Magnetic skyrmions: Advances in physics and potential applications. *Nat Rev. Mater.* **2**, 1–15 (2017).

25. D. Foster, C. Kind, P. J. Ackerman, J.-S. B. Tai, M. R. Dennis, I. I. Smalyukh, Two-dimensional skyrmion bags in liquid crystals and ferromagnets. *Nat. Phys.* **15**, 655–659 (2019).

26. M. Król, H. Sigurdsson, K. Rechcińska, P. Oliwa, K. Tyszka, W. Bardyszewski, A. Opala, M. Matuszewski, P. Morawiak, R. Mazur, W. Piecek, P. Kula, P. G. Lagoudakis, B. Piętka, J. Szczytko, Observation of second-order meron polarization textures in optical microcavities. *Optica* **8**, 255–261 (2021).

27. S. Donati, L. Dominici, G. Dagvadorj, D. Ballarini, M. De Giorgi, A. Bramati, G. Gigli, Y. G. Rubo, M. H. Szymańska, D. Sanvitto, Twist of generalized skyrmions and spin vortices in a polariton superfluid. *Proc. Natl. Acad. Sci. U.S.A.* **113**, 14926–14931 (2016).

28. C. He, Y. Shen, A. Forbes, Towards higher-dimensional structured light. *Light Sci. Appl.* **11**, 205 (2022).

29. S. Tsesses, E. Ostrovsky, K. Cohen, B. Gjonaj, N. H. Lindner, G. Bartal, Optical skyrmion lattice in evanescent electromagnetic fields. *Science* **361**, 993–996 (2018).

30. L. Du, A. Yang, A. V. Zayats, X. Yuan, Deep-subwavelength features of photonic skyrmions in a confined electromagnetic field with orbital angular momentum. *Nat. Phys.* **15**, 650–654 (2019).

31. Y. Shen, C. He, Z. Song, B. Chen, H. He, Y. Ma, J. A. J. Fells, S. J. Elston, S. M. Morris, M. J. Booth, A. Forbes, Topologically controlled multiskyrmions in photonic gradient-index lenses. *Phys. Rev. Appl.* **21**, 024025 (2024).

32. Y. Shen, E. C. Martínez, C. Rosales-Guzmán, Generation of optical skyrmions with tunable topological textures. *ACS Photonics* **9**, 296–303 (2022).

33. I. Nape, K. Singh, A. Klug, W. Buono, C. Rosales-Guzman, A. McWilliam, S. Franke-Arnold, A. Kritzinger, P. Forbes, A. Dudley, Revealing the invariance of vectorial structured light in complex media. *Nat. Photonics* **16**, 538–546 (2022).

34. Y. Shen, Q. Zhang, P. Shi, L. Du, X. Yuan, A. V. Zayats, Optical skyrmions and other topological quasiparticles of light. *Nat. Photonics* **18**, 15–25 (2024).

35. A. A. Wang, Y. Ma, Y. Zhang, Z. Zhao, Y. Cai, X. Qiu, B. Dong, C. He, Unlocking new dimensions in photonic computing using optical skyrmions. arXiv:2407.16311 [physics.optics] (2024).

36. S. Gao, F. C. Speirits, F. Castellucci, S. Franke-Arnold, S. M. Barnett, J. B. Götte, Paraxial skyrmionic beams. *Phys. Rev. A* **102**, 053513 (2020).

37. A. A. Wang, Z. Zhao, Y. Ma, Y. Cai, R. Zhang, X. Shang, Y. Zhang, J. Qin, Z.-K. Pong, T. Marozsák, B. Chen, H. He, L. Luo, M. J. Booth, S. J. Elston, S. M. Morris, C. He, Topological protection of optical skyrmions through complex media. *Light Sci. Appl.* **13**, 314 (2024).

38. B. Göbel, I. Mertig, O. A. Tretiakov, Beyond skyrmions: Review and perspectives of alternative magnetic quasiparticles. *Phys. Rep.* **895**, 1–28 (2021).

39. N. Nagaosa, Y. Tokura, Topological properties and dynamics of magnetic skyrmions. *Nat. Nanotechnol.* **8**, 899–911 (2013).

40. D. H. Goldstein, *Polarized Light* (CRC Press, 2017).

41. R. M. A. Azzam, Stokes-vector and Mueller-matrix polarimetry. *J. Opt. Soc. Am. A* **33**, 1396–1408 (2016).

42. D. H. Goldstein, Mueller matrix dual-rotating retarder polarimeter. *Appl. Opt.* **31**, 6676–6683 (1992).

43. P. Shukla, A. Awasthi, P. K. Pandey, A. Pradhan, "Discrimination of normal and dysplasia in cervix tissue by Mueller matrix analysis," in *Biomedical Applications of Light Scattering II* (SPIE, 2008), vol. 6864, pp. 248–255.

44. C. He, H. He, J. Chang, Y. Dong, S. Liu, N. Zeng, Y. He, H. Ma, Characterizing microstructures of cancerous tissues using multispectral transformed Mueller matrix polarization parameters. *Biomed. Opt. Express* **6**, 2934–2945 (2015).

45. Y. Wang, H. He, J. Chang, C. He, S. Liu, M. Li, N. Zeng, J. Wu, H. Ma, Mueller matrix microscope: A quantitative tool to facilitate detections and fibrosis scorings of liver cirrhosis and cancer tissues. *J. Biomed. Opt.* **21**, 071112 (2016).

46. Y. Dong, J. Qi, H. He, C. He, S. Liu, J. Wu, D. S. Elson, H. Ma, Quantitatively characterizing the microstructural features of breast ductal carcinoma tissues in different progression stages by Mueller matrix microscope. *Biomed. Opt. Express* **8**, 3643–3655 (2017).

47. J. Chang, H. He, Y. Wang, Y. Huang, X. Li, C. He, R. Liao, N. Zeng, S. Liu, H. Ma, Division of focal plane polarimeter-based 3× 4 Mueller matrix microscope: A potential tool for quick diagnosis of human carcinoma tissues. *J. Biomed. Opt.* **21**, 056002 (2016).

48. L. Deng, Z. Fan, B. Chen, H. Zhai, H. He, C. He, Y. Sun, Y. Wang, H. Ma, A dual-modality imaging method based on polarimetry and second harmonic generation for characterization and evaluation of skin tissue structures. *Int. J. Mol. Sci.* **24**, 4206 (2023).

49. J. Qi, C. He, D. S. Elson, Real time complete Stokes polarimetric imager based on a linear polarizer array camera for tissue polarimetric imaging. *Biomed. Opt. Express* **8**, 4933–4946 (2017).

50. H. He, C. He, J. Chang, D. Lv, J. Wu, C. Duan, Q. Zhou, N. Zeng, Y. He, H. Ma, Monitoring microstructural variations of fresh skeletal muscle tissues by Mueller matrix imaging. *J. Biophotonics* **10**, 664–673 (2017).

51. C. He, H. He, X. Li, J. Chang, Y. Wang, S. Liu, N. Zeng, Y. He, H. Ma, Quantitatively differentiating microstructures of tissues by frequency distributions of Mueller matrix images. *J. Biomed. Opt.* **20**, 105009 (2015).

52. Z. Zhang, R. Hao, C. Shao, C. Mi, H. He, C. He, E. Du, S. Liu, J. Wu, H. Ma, Analysis and optimization of aberration induced by oblique incidence for in-vivo tissue polarimetry. *Opt. Lett.* **48**, 6136–6139 (2023).

53. Z. Zhang, C. Shao, H. He, C. He, S. Liu, H. Ma, Analyzing the influence of oblique incidence on quantitative backscattering tissue polarimetry: A pilot *ex vivo* study. *J. Biomed. Opt.* **28**, 102905 (2023).

54. J. Chang, H. He, C. He, Y. Wang, N. Zeng, R. Liao, H. Ma, Optimization of GRIN lens Stokes polarimeter. *Appl. Opt.* **54**, 7424–7432 (2015).

55. C. Shao, B. Chen, H. He, C. He, Y. Shen, H. Zhai, H. Ma, Analyzing the influence of imaging resolution on polarization properties of scattering media obtained from Mueller matrix. *Front. Chem.* **10**, 936255 (2022).

56. C. He, J. Lin, J. Chang, J. Antonello, B. Dai, J. Wang, J. Cui, J. Qi, M. Wu, D. S. Elson, P. Xi, A. Forbes, M. J. Booth, Full Poincaré polarimetry enabled through physical inference. *Optica* **9**, 1109–1114 (2022).

57. V. V. Marenko, T. V. Molebnaya, Optimization of Stokes polarimeters employing a measurement of 4 intensities. *Sov. J. Opt. Technol.* **57**, 452–455 (1990).

58. D. S. Sabatke, M. R. Descour, E. L. Dereniak, W. C. Sweatt, S. A. Kemme, G. S. Phipps, Optimization of retardance for a complete Stokes polarimeter. *Opt. Lett.* **25**, 802–804 (2000).

59. R. M. A. Azzam, I. M. Elminyawi, A. M. El-Saba, General analysis and optimization of the four-detector photopolarimeter. *J. Opt. Soc. Am. A* **5**, 681–689 (1988).

60. R. M. A. Azzam, Arrangement of four photodetectors for measuring the state of polarization of light. *Opt. Lett.* **10**, 309–311 (1985).

61. R. A. Chipman, "Polarization Aberrations (Thin Films)," thesis, The University of Arizona, Tucson, AZ (1987).

62. R. Ossikovski, Interpretation of nondepolarizing Mueller matrices based on singular-value decomposition. *J. Opt. Soc. Am. A Opt. Image Sci. Vis.* **25**, 473–482 (2008).

63. J. D. Muñoz-Bolaños, P. Rajaeipour, K. Kummer, M. Kress, C. Ataman, M. Ritsch-Marte, A. Jesacher, Confocal Raman microscopy with adaptive optics. *ACS Photonics* **12**, 176–184 (2024).

64. M. Veettikazhy, J. Nylk, F. Gasparoli, A. Escobet-Montalbán, A. K. Hansen, D. Marti, P. E. Andersen, K. Dholakia, Multi-photon attenuation-compensated light-sheet fluorescence microscopy. *Sci. Rep.* **10**, 8090 (2020).
